# Fast-track protocols for patients undergoing spine surgery: a systematic review

**DOI:** 10.1186/s12891-022-06123-w

**Published:** 2023-01-23

**Authors:** Deyanira Contartese, Francesca Salamanna, Silvia Brogini, Konstantinos Martikos, Cristiana Griffoni, Alessandro Ricci, Andrea Visani, Milena Fini, Alessandro Gasbarrini

**Affiliations:** 1 Surgical Sciences and Technologies, IRCCS Istituto Ortopedico Rizzoli, 40136 Bologna, Italy; 2 Spine Surgery, IRCCS Istituto Ortopedico Rizzoli, Bologna, Italy; 3 Anesthesia-resuscitation and Intensive care, IRCCS Istituto Ortopedico Rizzoli, Bologna, Italy; 4 Scientific Direction, IRCCS Istituto Ortopedico Rizzoli, Bologna, Italy

**Keywords:** Fast-track, Spine surgery, Pre-, Intra- and postoperative elements, Lengths of stay, Pain

## Abstract

**Background context:**

Fast-track is an evidence-based multidisciplinary strategy for pre-, intra-, and postoperative management of patients during major surgery. To date, fast-track has not been recognized or accepted in all surgical areas, particularly in orthopedic spine surgery where it still represents a relatively new paradigm.

**Purpose:**

The aim of this review was provided an evidenced-based assessment of specific interventions, measurement, and associated outcomes linked to enhanced recovery pathways in spine surgery field.

**Methods:**

We conducted a systematic review in three databases from February 2012 to August 2022 to assess the pre-, intra-, and postoperative key elements and the clinical evidence of fast-track protocols as well as specific interventions and associated outcomes, in patients undergoing to spine surgery.

**Results:**

We included 57 full-text articles of which most were retrospective. Most common fast-track elements included patient’s education, multimodal analgesia, thrombo- and antibiotic prophylaxis, tranexamic acid use, urinary catheter and drainage removal within 24 hours after surgery, and early mobilization and nutrition. All studies demonstrated that these interventions were able to reduce patients’ length of stay (LOS) and opioid use. Comparative studies between fast-track and non-fast-track protocols also showed improved pain scores without increasing complication or readmission rates, thus improving patient’s satisfaction and functional recovery.

**Conclusions:**

According to the review results, fast-track seems to be a successful tool to reduce LOS, accelerate return of function, minimize postoperative pain, and save costs in spine surgery. However, current studies are mainly on degenerative spine diseases and largely restricted to retrospective studies with non-randomized data, thus multicenter randomized trials comparing fast-track outcomes and implementation are mandatory to confirm its benefit in spine surgery.

**Supplementary Information:**

The online version contains supplementary material available at 10.1186/s12891-022-06123-w.

## Introduction

Spine surgery is performed to correct spinal pathologies that cause pain and instability in both adult and pediatric patients and is one of the fastest expanding surgical specialties in the world [1–3]. Such procedures are commonly associated with severe postoperative pain, significant blood loss, functional limitation, and potential postoperative complications, due to the invasiveness of the surgery and prolonged hospitalization. In this regard, in recent years, clinical pathway and care methods emerged associated with the concept of fast-track programs. Fast-track surgery procedures, also identified as Enhanced Recovery After Surgery (ERAS), were first introduced in the 1990s by Henrik Kehlet [4]. The procedure consists of an evidence-based approach of care with the involvement of a multidisciplinary team made up of surgeons, nursing, anesthesiologists, physiatrists, physiotherapists and nutritionists, designed to prepare patients and reduce the impact of surgery, allowing them to recover more rapidly [4]. These programs aim to reduce stress related to surgery focusing on patient’s psychological well-being and the early mobilization, resulting in a rapid recovery and, consequently, a shorter length of hospital stay (LOS) [5]. LOS reduction leads, in turn, to a lower risk of infections and adverse events as well as to a reduction of the intraoperative complications and health care cost [6]. The procedures manage the patients care into a multimodal and multidisciplinary approach that include patient specific education, optimization and information on the pre-, intra-, and postoperative steps, improvements in surgical and anesthetic techniques, advances in postoperative multimodal analgesia, early rehabilitation and ambulation, early food intake, and discharge within 24 hours post-surgery [7, 8]. In the last few years, fast-track programs are successfully developing and are always undergoing improvement in several areas of orthopedic research and surgery. Particularly, there are several evidence to support the use of enhanced recovery pathways for patients undergoing to hip and/or knee orthopedic surgery. Although this type of pathways has several advantages and represents the standard of care in many surgical areas, to date, the clinical effectiveness of fast-track procedures has not been regularly recognized or accepted for all orthopedic field and there is still work to be done particularly in spine surgery [9–13]. Existing fast-track spine protocols are still in the early stage and vary significantly in their pre-, intra-, and postoperative elements, rendering difficult to assess their real effectiveness, farther there remains a lack of consensus on which specific elements may be relatively more effective. Thus, to highlight the most recent improvement in the pre-, intra-, and postoperative fast-track components and their clinical evidence in patients undergoing different spine surgery, we carried out a systematic review in order to provide an evidenced-based assessment of specific interventions, measurement, and associated outcomes linked to enhanced recovery pathways in spine surgery field.

## Methods

### Eligibility criteria

The PICOS model (population, intervention, comparison, outcomes, study design) was used to design this study: (1) studies that considered patients undergoing spine surgery (Population) submitted to, (2) fast-track protocols (Interventions), (3) with or without a comparison group (standard protocol) (Comparisons), (4) that reported pre-, intraoperative, and postoperative key components and clinical outcomes of the fast-track interventions (Outcomes), in (5) randomized, retrospective, and prospective e studies (Study design). Studies from February 1, 2012, to August 1, 2022, were included in this review if they met the PICOS criteria. We excluded studies that evaluated (1) surgeries other than spine, (2) patients undergoing spine surgery with other concomitant severe pathological conditions (i.e. tumor, metastases), and (3) articles with incomplete outcomes or data. Additionally, we excluded reviews, case reports or series, letters, comment to Editor, in vivo and in vitro studies, pilot studies, meta-analysis, editorials, protocols and recommendations, guidelines, and articles not written in English.

### Search strategies

Our literature review involved a systematic search conducted in August 2022. We performed our review according to the Preferred Reporting Items for Systematic Reviews and Meta-Analyses (PRISMA) statement [14]. The search was carried out on three databases: PubMed, Scopus, and Web of Science Core Collection. The following combination of terms was used (spine disease OR spine surgery) AND (fast-track OR enhanced recovery after surgery OR enhanced recovery programs), and for each of these terms, free words, and controlled vocabulary specific to each bibliographic database were combined using the operator “OR”. The combination of free-vocabulary and/or Medical Subject Headings (MeSH) terms for the identification of studies in PubMed, Scopus and Web of Science Core Collection were reported in Table 1 (Supplemental Material).

**Table 1:** Basic characteristics of included literatures studies on spine surgery

Ref.	Study design	Patients number, age (years) and gender (%)	Comparative analyses (Yes/No)	Surgery (indication and operation)	Spine level	Comorbidities	ICU LOS (days)	LOS (days)	Complications	Readmission and reoperatin rates	Follow-up	Outcomes/ Endpoints
Adeyemo et al. 2021a [15]	Retrospective	124 patients: -Fast-track group (*n* = 67, mean age 68.49 ± 8.72, 60% females); −Non-fast-track group (*n* = 57, mean age 69.7 ± 8.23, 67% females)	Yes	Thoraco-lumbar-pelvic fusion (open approach with posterior osteotomies, and pedicle screw fixation) for adult degenerative scoliosis	> 4	Osteoporosis	1.78 ± 2.85	7 ± 3.88	5.97% urinary retention, 1.49% constipation, 5.97% motor block, 4.48% arrhythmia, 1.49% delirium, 1.49% pneumonia	2.98% 90 days inpatient readmission rate	90 days	↑LOS (7 ± 3.88 vs. 5.82 ± 1.97 days), ↓opioid consumption (248.05 mg vs. 314.05 mg), urinary retention (5.97% vs. 19.3%), constipation (1.49% vs. 31.57%), motor block (5.97% vs. 15.79%), 90 days inpatient readmission rate (2.98% vs. 28.07%) EBL (1284.84 ml vs. 1691.8 ml) in fast-track group vs. non-fast-track group. =operative time, anesthesia duration, ICU LOS, 30 days ER visit rate, other complications
Adeyemo et al. 2021b [16]	Retrospective	83 patients: -Fast-track group (*n* = 46, mean age 70.22 ± 7.56, 59% females); -Non-fast-track group (*n* = 37, mean age 68.47 ± 9.16, 83,8% females)	Yes	Thoraco-lumbar-pelvic fusion (open approach with multiple-level posterior osteotomies, and pedicle screw and rod fixation) for adult degenerative scoliosis	T8-T9, T11	NR	1.96 ± 2.95	5.98 ± 2.65	6.52% urinary retention, 26.09% constipation, 2.17% pruritus, 10.87% cardiac arrhythmia, 2.17% delirium, 4.35% pneumonia, 10.42% motor block	0% 30 days inpatient readmission rate	6 months	↓Urinary retention (6.52% vs. 27.03%), constipation (26.09% vs. 62.16%) in fast-track group vs. non-fast-track group. =LOS, ICU LOS, operative time, anesthesia duration, EBL, 30 day inpatient readmission rate, 30 day ER visit rate, pain score, opioid consumption, ambulation distance, complications (pruritus, cardiac arrhythmia, delirium, pneumonia, motor block)
Angus et al. 2019 [17]	Retrospective	626 patients: -Fats-track group (*n* = 214, mean age 55.3, 129 females); −Non-fast-track group (*n* = 412, mean age 50.5, 135 females)	Yes	Elective surgery, PLIF (with/without bone grafting) for adult complex degenerative spinal deformity or multilevel adolescent scoliosis correction	> 1	NR	NR	5.2 and 8 days	NR	1.9% 30 days readmissions	2 years	↑Patient satisfaction (100% vs. 84%) and ↓LOS (5.2 vs. 7 days for complex fixation, and 8 vs. 11 days for degenerative scoliosis correction), complications, 30 days readmissions (1.9% vs. 2.1%) in fast-track group vs. non-fast-track group. =levels fused
Brusko et al. 2019 [18]	Retrospective	97 adult patients: -Fast-track group (n = 57, mean age 65.5 ± 9.3, 24 females); −Non-fast-track group (*n* = 40, mean age 68.1 ± 9.9, 20 females)	Yes	Elective, posterior lumbar fusion (open procedures and MIS with percutaneous pedicle screws)	1- to 3	NR	NR	2.9 ± 1.9 days	NR	NR	6 months	↓LOS (2.9 ± 1.9 vs. 3.8 ± 1.8 days), pain (at day 1, 4.2 ± 3.2 vs. 6.0 ± 3.2), oxycodone-acetaminophen consumption (at day 0, 408.0 ± 527.2 vs. 1094.7 ± 847.6 mg; at day 1, 1320.0 ± 1026.4 vs. 1708.4 ± 819.6 mg; at day 3, 1500.1 ± 778.5 mg vs. 2105.4 ± 1090.6 mg; during LOS, 2729.5 ± 4594.3 mg vs. 5230.3 ± 3920.3 mg), meperidine consumption (8.8 ± 32.9 vs. 44.7 ± 87.5 mg), IV pain medication (1.6 ± 1.2 vs. 2.0 ± 1.1 days), ondansetron consumption (2.81 ± 4.3 vs. 6.0 ± 10.5 mg) and ↑levels fused (92 vs. 62), hydromorphone consumption, distance ambulated (at day 1, 109.4 ± 130.4 vs. 41.4 ± 62.0 ft) in fast-track group vs. non-fast-track group
Carr et al. 2019 [19]	Retrospective	932 patients: -Fast-track group (*n* = 620, mean age 60 ± 13, 322 females); −Traditional care group (*n* = 183, mean age 61 ± 14, 103 females); -No pathway care group (*n* = 129, mean age 58 ± 13, 72 females)	Yes	Elective spine surgery (arthrodesis with instrumentation anterior > 2 levels or posterior > 3 levels, corpectomy in cervical, thoracic, or lumbar region, pelvic fixation) for adult spinal deformity	≥4 (nonrevision surgery), ≥3 (revision surgery)	Poor functional status, daily home oxygen, CPAP/BiPAP, unstable angina, pacemaker, automatic implantable cardiac defibrillator or ventricular assist device, pulmonary hypertension, diabetes with Hb A1c > 8%, hypertension or arrhythmias, coagulation disorders or patients receiving anticoagulants and antiplatelets	1.8 days	5.4 days	NR	NR	NR	↑Operative time in traditional care group vs. fast-track group and no pathway care group, and = between fast-track group and no pathway care group. ↓LOS (5.4 vs. 8.2 and 8.0 days), ICU LOS (1.8 vs. 3.1 and 2.5 days), costs (by 29 and 11%) in fast-track group vs. traditional care group and no pathway care group
Chang et al. 2020 [20]	Retrospective	48 patients: -Fast-track group (*n* = 24, mean age 64.3 ± 11.59, 10 females); −Non-fast-track group (n = 24, mean age 60.1 ± 12.23, 13 females)	Yes	TLIF (mesh expandable cage, percutaneous pedicle screw placement and rod fixation, off-label use recombinant human BMP-2) for adult degenerative lumbar spine disease	L1–2, L2–3, L3–4, L4–5, L5–S1	Obesity and sarcopenia	NR	1.4 ± 1.13 days	1 infection at the interbody space (2 months after surgery)	NR	3, 6 and 12 months	↑Postoperative ODI, discharge at day 1 (79%) and ↓LOS (1.4 ± 1.13 vs. 4.0 ± 1.98 days), operation time (110.7 ± 21.23 vs. 154.8 ± 39.53 min), EBL (66.0 ± 37.24 vs. 121.4 ± 62.39 ml), oral and IV opioid consumption (22.8 ± 20.20 mg on day 0 and 21.6 ± 18.72 mg on day 1 vs. 38.1 ± 23.27 mg on day 0 and 44.3 ± 23.10 mg on day 1) in fast-track group vs. non-fast-track group
Chen et al. 2021 [21]	Retrospective	78 patients: -Fast-track OLIF group (*n* = 38, mean age 61.84 ± 6.20, 44.7% females); −Fast-track TLIF group (n = 40, mean age 61.15 ± 5.52, 42.5% females)	No	OLIF (with pedicle screw-rod internal fixation, interstitial approach) and single-level instrumented TLIF (with polyaxial pedicle screws and crescent-shaped interbody cage) for adult lumbar degenerative diseases (disc herniation, spinal stenosis, degenerative slippage I-II degrees, spondylolysis)	L4/L5	Hypertension or diabetes	NR	7.87 ± 1.04 days	NR	NR	6 and 12 months	↓EBL (59.53 ± 11.80 vs. 102.48 ± 14.22 ml), LOS (7.87 ± 1.04 vs. 9.23 ± 0.95 days) in OLIF group vs. TLIF group. =operative time, overall satisfaction. ↓red blood count, albumin, VAS score, ODI, and ↑CRP, D-dimer, JOA in postoperative vs. preoperative
Dagal et al. 2019 [22]	Retrospective	450 adult patients: -Fast-track group (*n* = 267, mean age 60 ± 12, 55.4% females); -Non-fast-track group (n = 183, mean age 61 ± 14, 56.2% females)	Yes	Major elective spine surgery, cervico-thoracic, thoracolumbar levels	NR	Anemia, dementia, depression, diabetes, hypertension, coronary artery disease, congestive heart failure, chronic kidney disease, chronic pulmonary disease, cerebrovascular accident, chronic opioid use (substance abuse), obstructive sleep apnea	1.4 days	3.6 days	1.1% pneumonia, 1.1% pulmonary embolism, 0.7% sepsis, 1.5% surgical site bleeding, 8.2% wound infection, 0.4% death	9.7% 30 days readmissions	30 days	↓LOS (3.6 vs. 6.3 days), ICU LOS (1.4 vs. 4.7 days), costs ($62.429 to $53.355), EBL (711 vs. 1066 ml), postoperative ICU admissions (48% vs. 60%) in fast-track vs. non-fast-track group. =anesthesia duration, complications, 30 days readmission
d’Astorg et al. 2020 [23]	Retrospective	386 patients: -Fast-track group (n = 193, mean age 46 ± 12); −Non-fast-track group (*n* = 193, mean age 46 ± 13)	Yes	Microdiscectomy and arthrodesis (1 or 2-level retroperitoneal ALIF, 1 to 3-level circumferential fusion with combined approach ALIF + posterior instrumentation, 1 to 3-level posterior fusion, anterior cervical fusion) for adult spinal deformities (herniated lumbar disc, single or multilevel lumbar stenosis)	1 to 3	NR	NR	2.6 days	1 cervicalgia after load carrying 3 weeks after cervical fusion, 1 suspicion of pulmonary embolism, 1 malaise following intolerance to tramadol (3 visits to A&E department)	1 readmission (cleaning of the surgical scar)	1 year (arthrodesis) and 3 months (microdiscectomy)	↑Satisfaction and ↓LOS (2.6 vs. 4.4 days), pain VAS, ODI score in fast-track group vs. non-fast-track group. =complications, readmission
Debono et al. 2019 [24]	Retrospective	3483 patients: -Fast-track group (*n* = 1920: 202 ALIF, mean age 46.3 ± 10.7, 49% females; 612 ACDF, mean age 48.7 ± 8.7, 49% females; 1106 posterior fusion, mean age 56.1 ± 10.2, 50.9% females); −Non-fast-track group (*n* = 1563: 159 ALIF, mean age 44.5 ± 8.6, 56.6% females; 749 ACDF, mean age 47.6 ± 9.9, 45.6% females; 655 posterior fusion, mean age 53.8 ± 14.3, 49.7% females)	Yes	Elective spine surgery (retroperitoneal ALIF, ACDF with anterior approach, posterior or posterolateral fusion as PLIF and TLIF, with PEEK cages or plates) for adult degenerative conditions	NR	NR	NR	3.33 ± 0.8 days (ALIF), 1.3 ± 0.7 days (ACDF), 4.8 ± 2.3 days (posterior fusion)	11.4% for ALIF (medical 3.5%, wound 3.5%, neurological 1.5%, implants 1.0%, urinary tract infection 2.0%), 8.2% for ACDF (medical 2.1%, cervical approach 2.8%, implants 2.8%, infection 0.5%), 10.9% for posterior fusion (medical 2.6%, wound 2.5%, neurological 1.4%, dural leakage 1.7%, implants 2.0%, urinary tract infection 0.6%)	3.0% 90 days rehospitalization and 1.5% revision surgery for ALIF, 1.5% 90 days rehospitalization and 0.8% revision surgery for ACDF, 6.1% 90 days rehospitalization and 3.7% revision surgery for lumbar fusions	90 days	↓LOS (3.33 ± 0.8 vs. 6.06 ± 1.1 days for ALIF, 1.3 ± 0.7 vs. 3.08 ± 0.9 for ACDF, 4.8 ± 2.3 vs. 6.7 ± 4.8 for posterior fusion) in fast-track group vs. non-fast-track group. =complications, 90 days rehospitalization rate or revision rate for ALIF and ACDF. =90 days rehospitalization rate for posterior fusion. ↓complications (10.9% vs. 14.8%), surgical revision rate (3.7% vs. 6.1%) for lumbar fusions
Debono et al. 2021 [25]	Retrospective	404 patients: -Fast-track group (n = 202, mean age 48.5 ± 10.6, 49% females); −Non-fast-track group (*n* = 202, mean age 48.7 ± 9.2, 47.5% females)	Yes	ACDF (anterior approach, PEEK cages alone or with plates) for adult radiculopathy with disc prolapse either hard (osteophytic) or soft	NR	NR	NR	1.40 ± 0.6 days	6.9% overall complications, 3.5% major complications (new neurological deficit 2.5%, neck hematoma 1.0%), 3.5% minor complications (dysphagia/dysphonia 2.5%, hardware failure 0.5%, surgical site infection 0.5%)	0% 30 days readmission, 0% 30- to 90 days readmission, 1.0% 90 days reoperation	30 and 90 days, 12 months	↓LOS (1.40 ± 0.6 vs. 2.96 ± 1.35 days) in fast-track group vs. non-fast-track group. =satisfaction, complications (overall, major, minor), 30 days readmission, 30- to 90 days readmission, 90 days reoperation
DeVries et al. 2020 [26]	Retrospective	244 patients: -Fast-track group (*n* = 131, mean age 15.3 ± 1.9, 78.6% females); −Non-fast-track group (*n* = 113, mean age 15.2 ± 2.0, 77.0% females)	Yes	PIF for AIS	NR	NR	NR	3.4 days	50% (2/4) wound complications (surgical site drainage), 50% (2/4) wound dehiscence	66.6% (2/3) screw misplacement and/or removal, 33.3% (1/3) deep wound infection requiring irrigation and debridement, 40.0% (4/10) constipation, 20.0% (2/10) syncope, 10.0% (1/10) pain, 30.0% (3/10) other	30 days	↓LOS (3.4 vs. 5.2 days), patient-controlled analgesia discontinuation (51.7 vs. 62.0 h), catheter discontinuation (1.9 ± 0.3 vs. 2.4 ± 0.6 days), standing initiated (1.0 ± 0.09 vs. 1.9 ± 0.6 days), walking initiated (1.9 ± 0.3 vs. 3.0 ± 0.9 days), and ↑curve magnitude (67.5 ± 13.3° vs. 62.3 ± 10.8°), curve correction (45.8 ± 13.8° vs. 38.2 ± 12.1°) in fast-track group vs. non-fast-track group. =complications, 30 days readmission, 30 days reoperation, 30 days visit, EBL
Duojun et al. 2021 [27]	Retrospective	120 patients: -Fast-track group (n = 60, mean age 47.92 ± 5.89, 28 females); −Non-fast-track group (*n* = 60, mean age 48.60 ± 5.80, 31 females)	Yes	PETD for adult single-level LDH	L4/5	Obesity or intervertebral foraminal stenosis	NR	3.47 ± 1.14 days	1 nerve damage, 1 incision infection, 2 lumbar and leg pain, 1 respiratory infection, 1 gastrointestinal reactions	NR	NR	↓LOS (3.47 ± 1.14 vs. 5.65 ± 1.39 days), VAS pain score (2.25 ± 0.82 vs. 3.33 ± 0.60 at day 1, 1.87 ± 0.50 vs. 3.07 ± 0.66 at day 2, 1.47 ± 0.54 vs. 2.25 ± 0.47 at day 3) in fast-track group vs. non-fast-track group. =complications, ODI, operative time, costs
Feng et al. 2019 [28]	Retrospective	74 patients: -Fast-track group (*n* = 44, mean age 61 ± 10, 63.6% females); −Non-fast-track group (n = 30, mean age 59 ± 9, 70% females)	Yes	MIS-TLIF (ipsilateral side facetectomy, and interbody fusion with unilateral access, bilateral MIS decompression with unilateral approach, pedicle screws percutaneous via bilateral approaches) for adult lumbar spinal stenosis, spondylolisthesis, degenerative lumbosacral spine diseases, radiculopathy, or neurogenic claudication	L3–4, L4–5, L5-S1	Diabetes mellitus, chronic cardiovascular disease	NR	5 days	4.5% (1 cage migration without symptoms, 1 epidural hematoma with radiculopathy)	0 30 days readmission, 1 30 days reoperation	30 days	↓LOS (5 vs. 7 days), costs, EBL (100 vs. 150 ml), operative time (206 vs. 228 min), IV fluid volume (1625 vs. 1827 ml), drainage at day 1–3 (85.5 vs. 160 mL) in fast-track group vs. non-fast-track group. =complications, 30 days readmission and reoperation rates
Flanders et al. 2020 [29]	Retrospective	1290 adult patients: -Fast-track group (*n* = 1141, mean age 61.5 ± 13.4, 533 females); −Non-fast-track group (*n* = 149, mean age 61.9 ± 12.1, 72 females)	Yes	Elective spine and peripheral nerve surgeries (cervical/thoracic/lumbar laminectomy and/or instrumented fusion, anterior cervical discectomy and fusion, combined anterior/posterior surgeries, and peripheral nerve procedures, brachial plexus surgery, ulnar and median nerve decompression, and common peroneal nerve surgery)	1–3	Chronic obstructive pulmonary disease, obstructive sleep apnea	44.9%	3.4 days	17.7%	6.4% 30 days readmission, 7.4% 90 days readmission	1, 3, 6 and 18 months	↓Opioids use (38.6% vs. 70.5% at 1 month, 36.5% vs. 70.9% at 3 months, 23.6% vs. 51.9% at 6 months), patient-controlled analgesia use (1.4% vs. 61.6%), LOS (3.4 vs. 3.9 days), ICU admissions (44.9% vs. 78.9%), likely to have an indwelling catheter while recovering in the inpatient ward (23.0% vs. 55.1%), nonopioid and ↑mobility at day 0 (63.5% vs. 20.7%), ambulation at day 0 (41.8% vs. 17.2%) in fast-track group vs. non-fast-track group. =satisfaction, complications, readmissions within 30 or 90 days
Fletcher et al. 2020 [30]	Retrospective	197 patients (13.2 ± 3.2 age, 110 females, 87 males): -Fast-track + LOS < 3 days group (*n* = 56); −Fast-track + LOS 3–7 days group (*n* = 1111); −Fast-track + LOS > 7 days group (*n* = 30)	No	PSF for NMS	1–3, 4–5	NR	1.0, 3.1, 5.6 days	3.6 h	Pulmonary (1.8, 14, 40%), neurologic deficits (2, 4, 7%), infection (2, 1, 9%), decubitus ulcers (0, 1, 4%), cut out/loosening/implant malplacement (0, 2, 0%)	Readmission (9, 17, 27%)	180 days	↓Fusion to pelvis (38% vs. 71 and 73%), levels fused (12.9 vs. 15.1 and 15.3), LOS (3.6 vs. 4.5 and 5.1 h), pulmonary complication (1.8% vs. 14 and 40%) in LOS < 3 days group vs. LOS 3–7 days and LOS > 7 days groups. =readmission, EBL, transfusion, complications, time ICU, required ICU
Fletcher et al. 2021 [31]	Prospective	276 patients: -Fast-track group (*n* = 203, mean age 14.3 ± 2.1, 78.8% females); −Non-fast-track group (*n* = 73, mean age 16.09 ± 2.1, 80.2% females)	Yes	PSF for AIS	NR	NR	NR	2.2 days	2 wound dehiscence, 1 constipation	Readmission, revision surgery	3–4 weeks	↓Major curve (54.0° vs. 62.0°), major curve correction (39.0° vs. 45.5°), LOS (2.2 vs. 4.8 days), operative time (2.8 vs. 4.8 h), EBL (240.0 vs. 500.0 ml), EBL/level (24.0 vs. 47.2 ml), EBL (6.4 vs. 13.3%EBV), levels fused (10.1 ± 2.6 vs. 11.4 ± 1.6), implant density (16.0 vs. 23.0), VAS at discharge (2.0 vs. 4.0), osteotomies (46% vs. 94%) in fast-track group vs. non-fast-track group
Garg et al. 2021 [32]	Retrospective	812 patients: -Fast-track group (*n* = 316, mean age 49.1 ± 11.7, 45% females); −Non-fast-track group (*n* = 496, mean age 50.3 ± 12.4, 46,8% females)	Yes	Elective lumbar spinal fusion at 1, 2, or 3 levels with posterior approach (TLIF with open or MIS techniques) for various adult lumbar spinal disorders (lumbar disc herniation, low-grade spondylolisthesis, lumbar canal stenosis, degenerative disc disease, facet joint cyst)	1–3	Secondary osteoporosis, diabetes	NR	2.94 days	Complications Clavien-Dindo grade (6.6% grade I, 3.2% grade II, 1.9% grade III)	2.2% 60 days readmission, 1.3% 60 days reoperation	4 weeks, 6 and 12 months	↓LOS (2.94 vs. 3.68 days), VAS score at 1 month (44 ± 10.8 vs. 49.8 ± 12.0), ODI score at 1 month (28 ± 12.8 vs. 31.6 ± 14.2) in fast-track group vs. non-fast-track group. =levels fused, EBL, operative time, complications, 60-day readmission, 60-day reoperation
Gong et al. 2021 [33]	Retrospective	91 patients: -Fast-track group (*n* = 46, mean age 55.2 ± 10.8, 30 females); −Non-fast-track group (*n* = 45, mean age 56.8 ± 8.9, 26 females)	Yes	PELIF for adult degenerative disc disease (degenerative spondylolisthesis, lumbar spinal canal stenosis, segmental instability, recurrent lumbar disc herniation, lumbar discogenic pain, isthmic spondylolisthesis)	1 or 2	Hypertension, diabetes mellitus, coronary artery disease, chronic obstructive pulmonary disease, asthma, liver disease	NR	3.1 ± 0.7 days	2	0 readmission	30 days	↓Opioid consumption (25.0 vs. 33.3), VAS score at day 1 (2.0 ± 0.6 vs. 2.6 ± 0.7) in fast-track group vs. non-fast-track group. =operative time, EBL, surgical dram drainage, LOS, cost, complication, 30 days readmission
He et al. 2020 [34]	Prospective	40 patients: -Fast-track + TXA group (n = 20, mean age 57.95 ± 12.44, 60% females); −Non-fast-track group (n = 20, mean age 57.9 ± 11.76, 45% females)	Yes	TLIF surgery (with pedicle screws, rods and cage filled with autogenous bone graft) for adult lumbar disc herniation, stenosis, or spondylolisthesis with unilateral radiculopathy	1 or 2	NR	NR	5.5 ± 2.0 days	1 superficial wound infection, 1 hypoproteinemia, 3 liver dysfunctions	NR	NR	↓EBL (91.50 ± 37.31 vs. 145 ± 108.7 ml), time to ambulation in fast-track group vs. non-fast-track group. =LOS, operative time, drainage, time for drainage removal, complications, Hb at day 1
Heo et al. 2019 [35]	Retrospective	69 patients: -Fast-track group (endoscopic TLIF, *n* = 23, mean age 61.4 ± 9.4, 69.6% females); −Non-fast-track group66 (microscopic TLIF, n = 46, mean age 63.5 ± 10.5, 58.6% females)	Yes	MIS-TLIF (with percutaneous biportal endoscopic approach and percutaneous pedicle screw insertion, cages and local autologous bone chips) for adult low-grade degenerative spondylolisthesis (grade 1), low-grade isthmic spondylolisthesis (grade 1), central stenosis with instability, and central stenosis with concomitant foraminal stenosis	L3–4, L4–5, L5-S1	NR	NR	NR	1 symptomatic epidural hematoma, 1 cage subsidence	0 readmission	Mean 13.4 ± 2.5 months	↓VAS score for preoperative back pain on day 1 and 2 (4.2 ± 1.0 vs. 4.9 ± 1.3 and 2.8 ± 0.5 vs. 4.2 ± 0.8), EBL (190.3 ± 31.0 vs. 289.3 ± 58.5 ml) and ↑operative time (152.4 ± 9.6122.4 ± 13.1 ml) in fast-track group vs. non-fast-track group. =VAS back and leg pain scores and ODI at final follow-up, complications and readmission rates, fusion rate
Ifrach et al. 2020 [36]	Prospective	564 adult patients: -Fast-track group (*n* = 504, mean age 73.2, 47% females); −Non-fast-track group (*n* = 60, mean age 73.5, 53.3% females)	Yes	Elective spine and peripheral nerve surgery (cervical/thoracic/lumbar laminectomy and/or instrumented fusion, ACDF, combined anterior-posterior surgeries, peripheral nerve procedures, brachial plexus surgery, ulnar and radial nerve decompression, carpal tunnel release, and common peroneal nerve surgery)	0–3 or 4+	Diabetes, hypertension, chronic obstructive pulmonary disease, mental health disorders, and substance abuse disorders, sleep apnea	NR	3.7 days	NR	NR	1 and 3 months	↓Opioid consumption (36.2% vs. 71.7% at 1 month, 33.0% vs. 80.0% at 3 months), patient-controlled analgesia (0.8% vs. 58.9%), catheters use (26.6% vs. 60.3%), LOS (3.7 vs. 4.3 days), pain and ↑mobilization at day 0 (60.0% vs. 10.0%), ambulation at day 0 (36.1% vs. 10.0%) in fast-track group vs. non-fast-track group. =mobilization and ambulation at day 1
Jazini et al. 2021 [37]	Retrospective	290 patients: -Fast-track group (*n* = 116, mean age 54.63 ± 13.05, 50% females); −Non-fast-track group (*n* = 174, mean age 54.56 ± 15.31, 52% females)	Yes	Lumbar fusion surgery ALIF and PIF for degenerative conditions	< 4	Stroke, DVT	NR	3.69 days	NR	NR	90 days	↓Pain scores at 3 months (2.89 vs. 3.57), in-hospital opioid consumption (374.43 vs. 781.25 MMEs), and ↑day ambulated (0.39 vs. 0.84), day catheter removed (1.14 vs. 1.44) in fast-track group vs. non-fast-track group. =90-day opioid consumption, EBL, operative time, LOS
Julien-Marsollier et al. 2020 [38]	Retrospective	163 patients (< 18 age): -Fast-track group (*n* = 81, mean age 15 ± 2, 81.5% females); −Non-fast-track group (*n* = 82, mean age 15.3 ± 1.8, 82.9% females)	Yes	Posterior fusion for AIS	NR	NR	NR	4 days	Opioid side effects (56.8% constipation, PONV), pain intensity, wound infection	NR	30 days	↓LOS (4 vs. 7 days), morphine consumption (25 and 35% at days 2 and 3), constipation at day 3 (56.8% vs. 73.2%), pain intensity at rest and movement at days 2 and 3 in fast-track group vs. non-fast-track group. =morphine consumption at day 1, PONV, wound infection
Kalinin et al. 2021 [39]	Prospective	53 patients: -Fast-track group (n = 24, mean age 58, 10 females); −Non-fast-track group (*n* = 29, mean age 55, 11 females)	Yes	Two-level transforaminal interbody fusion (dorsal decompression and stabilization surgeries) for polysegmental degenerative diseases of the lumbar spine (lower back pain and radicular clinical symptoms, involvement of two adjacent vertebral segments, and absence of improvement after conservative treatment for 6–8 weeks)	L2, L3, L4, L5, L6, S1	Diabetes, arterial hypertension, kidney diseases, lung diseases, coronary artery disease	NR	NR	1 bradycardia, 1 dizziness, 1 nausea, 1 venous thromboembolic complication, pseudoarthrosis	No re-hospitalization	18 months	↓Operative time (168 vs. 256 min), anesthesia time (185 vs. 270 min), EBL (75 vs. 180 ml), agents administered for anesthesia-0.005% fentanyl (20.0 vs. 31 ml), verticalization time (1 vs. 2 days), duration of inpatient treatment (9 vs. 10 days), pain, complication, ICU and ↑quality of life indicators, physical and psychological components of health in fast-track group vs. non-fast-track group
Kerolus et al. 2021 [40]	Retrospective	299 patients: -Fast-track group (*n* = 87, mean age 62.44 ± 11.66, 51 females); −Non-fast-track group (*n* = 212, mean age 60.17 ± 13.21, 116 females)	Yes	Elective single-level MIS-TLIF (with bilateral pedicle screw fixation) for degenerative disease	NR	NR	NR	3.13 ± 1.53 days	12.6% delirium, 48.3% urinary retention	0 90 days reoperation, 5.7% 30 days readmission	20 months	↓LOS (3.13 ± 1.53 vs. 3.71 ± 2.07 days), total daily average MME (50.55 ± 63.48 vs. 91.18 ± 99.76 MME), total admission MME (252.74 ± 317.38 vs. 455.91 ± 498.78), opioid consumption at day 1 (72.79 ± 70.52 vs. 177.60 ± 134.69 MME), at day 4 and onwards (21.37 ± 54.93 vs. 73.67 ± 262.34 MME), patient-controlled analgesia (29.9% vs. 86.8%), catheterization (48.3% vs. 65.6%) in fast-track group vs. non-fast-track group. =pain, opioid consumption at day 0, non-patient-controlled analgesia IV and oral opioids, delirium, operative time, 30 days readmission, 90 days reoperation
Kilic et al. 2019 [41]	Retrospective	120 patients: -Fast-track group (*n* = 60, mean age 50.43 ± 6.84, 30 females); −Non-fast-track group (*n* = 60, mean age 49.80 ± 6.04, 35 females)	Yes	Single-level lumbar microdiscectomy	NR	NR	NR	26.52 ± 5.16 h	NR	NR	NR	↓Operative time (78.50 ± 25.20 vs. 86.42 ± 18.39 min), EBL (93.17 ± 48.89 vs. 187.67 ± 47.37 ml), opioid administration (50 vs. 147.92 ± 22.69 μg), fluid administration (665.0 ± 233.49 vs. 2044.1 ± 401.38 ml), time to oral intake (2.88 ± 0.92 vs. 4.90 ± 1.08 h), time to mobilization (4.10 ± 0.95 vs. 7.20 ± 2.33 h), PONV (15.0% vs. 63.3%), analgesic required (13.3% vs. 100%), LOS (26.52 ± 5.16 vs. 30.10 ± 7.80 h), anesthesia cost (73.00 ± 24.93 vs. 270.42 ± 87.16TL), operation cost (1258.67 ± 39.89 vs. 1991.67 ± 67.12TL), VAS scores at 6 h (1.68 ± 1.40 vs. 4.03 ± 0.88) and at 12 h (1.12 ± 0.80 vs. 3.08 ± 0.90) in fast-track group vs. non-fast-track group
Kilic et al. 2020 [42]	Retrospective	174 patients: -Fast-track group (*n* = 86, mean age 54.79 ± 13.73, 53.4% females); −Non-fast-track group (*n* = 88, mean age 49.77 ± 16.96, 53.4% females)	Yes	Elective lumbar spine instrumentations for idiopathic lumbar scoliosis, degenerative spondylolisthesis, spinal canal stenosis	NR	Chronic cardiovascular disease, chronic pulmonary disease, diabetes mellitus	NR	31.24 ± 4.87 h	11.6% complications	1.48 ± 0.85 30 days readmission	30 days	↓EBL (204.42 ± 124.40 vs. 414.26 ± 237.64 ml), transfusion (1.08 ± 0.29 vs. 2.00 ± 0.92unit), first oral intake (4.34 ± 0.85 vs. 8.82 ± 3.41 h), first mobilization (13.80 ± 1.41 vs. 25.40 ± 3.13 h), LOS (31.24 ± 4.87 vs. 49.52 ± 5.96 h), pain scores at 12 h (1.84 ± 0.96 vs. 4.65 ± 1.41) and at 24 h (1.74 ± 0.81 vs. 4.48 ± 1.31), anesthesia cost (232.32 ± 19.44 vs. 533.86 ± 19.56TL), ICU cost (3726.51 ± 934.70 vs. 4994.09 ± 847.31TL), laboratory cost (279.30 ± 16.43 vs. 383.64 ± 18.39TL), radiology cost (271.98 ± 13.36 vs. 407.16 ± 49.31 TL) in fast-track group vs. non-fast-track group. =operative time, 30 days readmission, complication, surgery cost
Kim et al. 2021 [43]	Retrospective	40 patients: -Fast-track group (n = 20, mean age 65.7 ± 8.1, 65.0% females); −Non-fast-track group (*n* = 20, mean age 66.7 ± 9.6, 80% females)	Yes	≥5 levels of fusion to the pelvis with pedicle screws, rods, bone grafting for thoracolumbar adult deformity	L2-L5	Diabetes, osteoporosis, depression, hypertension, chronic pulmonary disease, chronic kidney disease	NR	4.5 ± 1.3 day	10% (2 dural tears)	20% (1 revision for a proximal junctional failure, 2 revisions for traumatic L5 pedicle fracture and proximal junctional failure)	90 days	↓EBL (920 ± 640 vs. 1437 ± 555 ml), ICU (0% vs. 30%), LOS (4.5 ± 1.3 vs. 7.3 ± 4.4 days), operative time (4.1 ± 1.2 vs. 5.0 ± 1.1 h), and ↑ambulation at day 1 (100% vs. 55%), EBL < 1200 mL (75% vs. 45%), procedure length < 4.5 h (66.7% vs. 33.3%) in fast-track group vs. non-fast-track group. =90 days readmission, complications, transfusion, discharge, drain and catheter discontinuation, levels fused
Lampilas et al. 2021 [44]	Retrospective	88 patients: -Fast-track group (n = 44, mean age 55.1 ± 15.8, 50% females); −Non-fast-track group (*n* = 44, mean age 55 ± 17.9, 38% females)	Yes	ALIF, ACF, lumbar release, LDH, cervical laminectomy	NR	NR	NR	3.3 ± 0.9 days	6 early unscheduled consultations (5 for pain, and 1 for postoperative neurologic deficit)	4.5% 90 days readmission (1 for pain resistant to home analgesia, 1 for cerebrospinal fluid leakage)	6 months	↓LOS (3.3 ± 0.9 vs. 6 ± 2.9 days), admission costs (5415 ± 1714 vs. 6302 ± 2303€) in fast-track group vs. non-fast-track group. =complications, 90 days readmission, total costs
Li et al. 2018 [45]	Retrospective	224 patients: -Fast-track group (n = 114, mean age 58.53 ± 10.71, 42.1% females); −Non-fast-track group (*n* = 110, mean age 56.88 ± 8.82, 39% females)	Yes	Cervical laminoplasty for degenerative multilevel spine compression, spinal canal stenosis	C3-C7	Diabetes mellitus, cardiovascular disease	NR	5.75 ± 2.46 days	3.51% C5 palsy, 4.39% incisional infection, 8.77% nausea and vomiting, 0.88% pulmonary infection, 1.75% urinary infection, 0.88% neurological deterioration, 0.88% epidural hematoma	NR	3 days	↓LOS (5.75 ± 2.46 vs. 7.67 ± 3.45 days), first assisted walking time (30.79 ± 14.45 vs. 65.24 ± 25.34), drains removal time (43.92 ± 7.14 vs. 48.85 ± 10.10 h), catheters removal time (24.76 ± 12.34 vs. 53.61 ± 18.16 h), first eating time (8.45 ± 2.94 vs. 21.64 ± 2.66 h), mean VAS score (2.72 ± 0.46 vs. 3.35 ± 0.46), maximum VAS score (3.76 ± 1.12 vs. 4.35 ± 1.15) in fast-track group vs. non-fat-track group. =operative time, EBL, first defecation time, complications, outbreak pain (VAS ≥ 5)
Li et al. 2020 [46]	Retrospective	Fast-track group, 260 patients: -Higher compliance group (*n* = 91, mean age 69.6 ± 4.4, 51 females); −Lower compliance group (*n* = 169, mean age 73.3 ± 7.1, 97 females)	No	Open posterior lumbar fusion surgery for lumbar stenosis with instability, scoliosis/ spondylolisthesis	1–2 or > 3	Hypertension, diabetes, ischemic heart disease, arrhythmias, gastrointestinal, chronic lung disease, Parkinson disease, depression	NR	11.8 ± 4.5 and 14.6 ± 6.1 days	40 complications (13 surgical site infection, 3 neurological deficit, 4 electrolyte abnormality, 4 pneumonia, 1 DVT/thrombophlebitis, 1 pulmonary embolism, 3 myocardial infarction, 2 urinary tract infection, 1 stroke, 1 sepsis, 7 delirium)	2 30 days readmission (surgical site infection), no re-operation or death	30 days	↓LOS (11.8 ± 4.5 vs. 14.6 ± 6.1 days), complications (8 vs. 32) in higher compliance group vs. lower compliance. =operative time, EBL, 30 days readmission
Li et al. 2021 [47]	Retrospective	127 patients: -Fast-track group (n = 60, mean age 73.6 ± 3.2, 63.3% females); −Non-fast-track (n = 67, mean age 74.3 ± 4.2, 59.7% females)	Yes	Open lumbar arthrodesis for lumbar stenosis	1–2 or > 3	Hypertension, diabetes, ischemic heart disease, arrhythmias, gastrointestinal, chronic lung disease, Parkinson disease, depression	NR	13.6 ± 4.0 days	8.3% complications (5: 2 surgical site infection, 1 electrolyte abnormality, 1 arrhythmia, 1 cerebrospinal fluid leakage)	NR	30 days	↓LOS (13.6 ± 4.0 vs. 15.6 ± 3.9 days), complications (8.3% vs. 20.9%), and ↑early ambulation (70.0% vs. 7.5%), early oral feeding (86.7% vs. 3.0%), early removal of catheter (80.0% vs. 14.9%), nutritional intervention (45.0% vs. 19.4%), VAS back at day 1 (3.8 ± 1.7 vs. 5.7 ± 2.3) and at day 2 (3.6 ± 1.9 vs. 4.5 ± 2.2) in fast-track group vs. non-fast-track group. =operative time, EBL
Nazarenko et al. 2016 [48]	Prospective	48 patients: -Fast-track group (n = 23, mean age 44.3, 39.1% females); −Non-fast-track group (*n* = 25, mean age 42.2, 44% females)	Yes	Microdiscectomy for lumbosacral spine herniated intervertebral discs	L1-L2, L3-L4, L4-L5, L5-S1	NR	NR	2.3 days	1 poor healing of wound	NR	1, 3 and 6 months	↓VAS pain at discharge (2.8 vs. 3.8) and at 1 month (1.7 vs. 2.6), ODI at discharge (11 vs. 19) and at 1 month (8 vs. 17), Roland-Morris scale at discharged (9 vs. 13) and at 1 month (8 vs. 11), LOS (2.3 vs. 3.8 days), and ↑satisfaction in fast-track group vs. non-fast-track group. =operative time, EBL
Rao et al. 2021 [49]	Retrospective	117 patients: -Fast-track group (*n* = 39, mean age 15.0 ± 2.4, 87.2% females); −Non-fast-track group (*n* = 78, mean age 14.3 ± 1.9, 83.3% females)	Yes	PSF for AIS	NR	NR	NR	3.8 ± 0.9 days	NR	0 readmission	NR	↓LOS (3.8 ± 0.9 vs. 4.6 ± 0.9 days), epidural h (11.8% vs. 16.0%), patient-controlled analgesia discontinuation (2 vs. 3 days), opioids use (2.2 ± 0.9 vs. 2.5 ± 1.1 mg IV morphine equivalents/kg), and ↑catheter removed by day 2 (95.0% vs. 80.8%), IV acetaminophen use (100% vs. 66.7%), oral acetaminophen (100% vs. 60.3%), ketorolac use (100% vs. 46.2%), ibuprofen use (48.7% vs. 6.4%) in fast-track group vs. non-fast-track group. =levels fused, operative time, EBL, pain score, 30 days readmission
Shaw et al. 2021 [50]	Retrospective	78 patients: -Fast-track + methadone group (n = 26, mean age 15.1 ± 1.9); −Fast-track group (no methadone, *n* = 52, mean age 14.9 ± 1.9)	No	PSF (with pedicle screw instrumentation) for AIS	NR	NR	NR	2.7 ± 0.7 and 3.1 ± 0.6 days	0 complications	2 30 days readmission	90 days	↓LOS (2.7 ± 0.7 vs. 3.1 ± 0.6 days), valium (11.3 ± 8.7 vs. 17.7 ± 11.7 mg) in fast-track + methadone group vs. fast-track alone group. =levels fused, operative time, EBL, opioid use, pain score
Smith et al. 2019 [51]	Retrospective	219 patients: -Fast-track group (*n* = 96, mean age 61.3 ± 13.3, 50.0% females); −Non-fast-track group (*n* = 123, mean age 60.3 ± 12.9, 56.9% females)	Yes	Lumbar spine fusion surgery	1–2	Coronary artery disease, hypertension, asthma, chronic obstructive pulmonary disease, diabetes mellitus-non-insulin dependent, diabetes mellitus-insulin dependent, history of cerebrovascular accident, anxiety, depression, kidney disease, liver disease, obstructive sleep apnea, substance abuse	NR	92.3 h	2.1% infections	NR	3, 6 and 12 months	↑Dexamethasone use (27% vs. 4.8%), methocarbamol use (62% vs. 44%), anticonvulsants use (67% vs. 22%), and ↓antiemetics use (24% vs. 40%), opioid use with patient-controlled analgesia after 24 h (0% vs. 7%), long-acting opioids use (5.2% vs. 14.6%), muscle relaxants (65.6% vs. 78.9%) in fast-track group vs. non-fast-track group. =LOS, mobility, complication, short-acting opioids use, pain score
Soffin et al. 2019b [52]	Retrospective	61 patients: -Fast-track + microdiscectomy group (*n* = 34, mean age 46, 50% females); −Fast-track + decompression group (*n* = 27, mean age 65, 48.4% females)	No	Lumbar microdiscectomy or decompression	1 and 1, 2 or 3	Diabetes mellitus, hypertension, coronary artery disease, chronic obstructive pulmonary disease	NR	285 and 298 min	0 complications	0 90 days readmission	90 days	↓LOS (285 vs. 298 min), operative time (48.8 ± 12.7 vs. 64.1 ± 28.6 min) in fast-track + microdiscectomy group vs. fast-track + decompression group. =EBL, IV fluid, opioids use
Soffin et al. 2019a [53]	Retrospective	33 patients: -Fast-track and ACDF group (n = 25, mean age 58, 80% females); −Fast-track and CDA group (n = 8, mean age 44, 75% females)	No	ACDF or CDA	1, 2, 3	Diabetes, hypertension, chronic obstructive pulmonary disease	NR	416 min	NR	No 90 days readmission	90 days	=Operative time, EBL, LOS, IV fluid, opioids use
Soffin et al. 2019c [54]	Retrospective	36 patients: -Fast-track + OFA group (n = 18, mean age 61.5 ± 18.92,44.4% females, 10 males); −Fast-track + OCA group (*n* = 18, mean age 60.14 ± 15.4, 44.4% females)	No	Elective lumbar decompression (laminectomy, laminotomy, and/or microdiscectomy)	NR	Hypertension, diabetes mellitus, hyperlipidemia, obstructive sleep apnea	NR	237 and 247 min	NR	NR	NR	↓Perioperative opioid use (2.43 ± 0.86 vs. 38.125 ± 6.11OMEs) in fast-track + OFA group vs. fast-track + OCA group. =LOS, operative time, pain score
Soffin et al. 2020 [55]	RCT	51 patients: -Fast-track group (n = 25, mean age 55 ± 18, 44% females); −Non-fast-track group (n = 26, mean age 54 ± 13, 69.2% females)	Yes	Primary lumbar fusion	1 or 2	Hypertension, asthma/pulmonary disease, coronary artery disease, obstructive sleep apnea	NR	2.8 days	32% nausea, 12% vomiting, 16% ileus, 4% delirium/confusion, 4% DVT/pulmonary embolus, 4% infection, 8% respiratory	NR	56 days	↑QoR40 scores at day 3 (179 ± 14 vs. 170 ± 16), and ↓time to first oral intake, pain score at day 1 (3 vs. 4), opioid use at 24 h (62 vs. 133) and at 48 h (30 vs. 75), IV patient-controlled analgesia duration (16 vs. 26 h), C-reactive protein at 3 days (6.1 vs. 15.9 mg·dl − 1) in fast-track group vs. non-fast-track group. =plasma biomarkers, complications, LOS, time to discharge from physical therapy
Staartjes et al. 2019 [56]	Prospective	Fast-track group: 2579 patients, mean age 48.5 ± 13.5, 45.9% females	No	Tubular microdiscectomy, 1-level robot-guided PLIF or TLIF, mini-open ALIF, or mini-open decompression for lumbar disc herniation, spinal stenosis, spondylolisthesis, facet cysts, or proven DDD	L1–2, L2–3, L3–4, L4–5, L5-S1	NR	NR	1.1 ± 1.2, 1.4 ± 0.7, 1.9 ± 0.6 days	4% complications	0.78% 30 days readmissions, 1.40% 60 days readmissions (67% unmanageable pain, 17% persistent CSF leakage with dizziness and orthostatic headache), 14% reoperation	6 weeks, 1 and 2 years	LOS = 1.1 ± 1.2 days; discharged at day 0 or 1 = 94%; discharged after day 1 = 85% (ALIF) and 52% (TLIF). ↓LOS (1.4 ± 0.7 vs. 1.9 ± 0.6 days) in ALIF vs. TLIF. ↑discharged at day 0 (98% vs. 22%) in discectomy vs. PLIF. ↑PROMs, D ODI, EQ-5D index, EQ-VAS, discharge at day 1 (from 90 to 96%), 1-night hospital stays (from 26 to 85%), and ↓operative time (from 38.8 ± 36.1 to 29.0 ± 22.8 min), complication, nursing costs (by 46.8%), LOS (from 2.4 ± 1.2 to 1.5 ± 0.3 days). =pain
Venkata et al. 2018 [57]	Prospective	Fast-track group: 237 patients (mean age: 57, 40% females)	No	Elective, open, non-instrumented lumbar and cervical spinal decompression and discectomy surgery for degenerative lumbar and cervical spinal conditions causing neural compression	1, 2, 3	NR	NR	< or > 24 h	1.6% disc prolapses, 0.8% hematomas	2.5% readmission (n = 7), reoperation (n = 6)	18 months	↓LOS: short stay = 12 patients (5%), ambulatory = 225 (95%) and day surgery after admission = 126 (53.2%)
Wang et al. 2017 [58]	Retrospective	Fast-track group: 42 patients (mean age 66.1 ± 11.7, 52% females)	No	1- or 2-level unilateral open TLIF (endoscopic decompression, expandable cage with allograft matrix, 2.1 mg rhBMP-2, bilateral pedicle percutaneous screws with 20 ml Exparel and bilateral rods) for spondylolisthesis or a severely degenerated disc with nerve root impingement, radiculopathy from neural compression, back pain from instability	L1–2, L2–3, L3–4, L4–5, L5-S1	NR	NR	1.29 ± 0.9 nights	1 cage displacement, 2 infection of interbody graft with sepsis, 1 atrial fibrillation, 1 upper-extremity DVT, 2 transient radiculitis	1 reoperation (graft migration at 2 months after surgery)	6 weeks, 3, 6, 12, and 24 months	Operative time = 94.6 ± 22.4 min; EBL = 66 ± 30 ml; LOS = 1.29 ± 0.9 nights. ↓ODI score (from 40 ± 13 to 17 ± 11)
Wang et al. 2020 [59]	Retrospective	190 patients: -Fast-track group (*n* = 95, 72.39 ± 6.12 age, 52.6% females); −Non-fast-track, (*n* = 95, mean age 70.81 ± 6.27, 57.8% females)	Yes	Lumbar fusion surgery for lumbar disk herniation or spinal stenosis	1 or 2	Hypertension, heart disease, diabetes, osteoporosis, gastrointestinal, psychological symptoms	NR	12.30 ± 3.03	1 spinal fluid leakage, 1 neurological	1 30 days readmission	30 days	↓LOS (12.30 ± 3.03 vs. 15.50 ± 1.88) in fast-track group vs. non-fast-track group. =operative time, levels fused, EBL, pain score, complication, mortality, 30 days readmission
Yang et al. 2020 [60]	Prospective	Fast-track group: 46 patients (mean age 14.3, 89.1% female)	No	PSF (with local autograft and allograft bone graft) for AIS	< or > L2	NR	NR	3.3 days	5% constipation	NR	15 days	LOS = 3.3 days: 1 patient discharge at day 2, 33 at day 3, 9 at day 4, 3 at day 5. Satisfaction on discharge at appropriate time = 80%, at discharge too early = 20%. ↓pain score (3.4 ± 1.6 vs. 4.7 ± 1.6) in appropriate group vs. too early group. =satisfaction, levels fused
Yang et al. 2020 [61]	Retrospective	72 patients: -Fast-track group (*n* = 51, mean age 70.1 ± 3.9, 28 females); −Non-fast-track group (n = 21, mean age 72.4 ± 5.4, 13 females)	Yes	TLIF (with interbody cage with autologous bone, pedicle screws and rods) for lumbar degenerative diseases (severe degenerative lumbar spinal stenosis, degenerative lumbar spondylolisthesis, lumbar disc herniation) and severe or progressive mechanical low back pain	L4–5	Hypertension, diabetes	NR	9.0 days	NR	NR	1 week, 2 years	↓Operative time (175.0 vs. 189.0 min), EBL (170.0 vs. 197.0 ml), LOS (9.0 vs. 12.0 days), NSAID use (37.50 vs. 45.00 mg), ambulation recovery time (1.0 vs. 2.0 day), VAS pain (2.0 vs. 3.0) at 3 days and 1 month, and ↑Barthel index at 3 days (65.0 vs. 30.0) and at 1 month (95.0 vs. 85.0) in fast-track group vs. non-fast-track group. =levels fused
Yang et al. 2021 [62]	Retrospective	79 patients: -Fast-track group (*n* = 35, mean age 14.6 ± 2.0, 27 females); −Non-fast-track group (n = 44, mean age 14.5 ± 2.1, 31 females)	Yes	PSF (with pedicle screw-rod system, autogenous local bone graft and allogeneic bone graft, without 3-column osteotomy) for AIS	NR	NR	NR	5.2 ± 1.6 days	2.9% complications	NR	1 year	↓Operative time (231.6 ± 34.7 vs. 290.9 ± 58.4 min), EBL (432.7 ± 201.1 vs. 894.3 ± 316.5 ml), allogeneic blood transfusion (3% vs. 33%), pain relief time (44.3 ± 33.5 vs. 70.5 ± 26.7 h), hemovac drainage (40.3 ± 24.8 vs. 691.7 ± 308.7 ml), drainage removal time (21.8 ± 9.8 vs. 60.4 ± 13.0 h), first ambulation time (23.9 ± 10.6 vs. 73.5 ± 18.3 h), LOS (5.2 ± 1.6 vs. 7.8 ± 1.5 days), PONV (14.3% vs. 34.1%) in fast-track group vs. non-fast-track group. =levels fused, Hb level, pain score, complications
Young et al. 2021 [63]	Retrospective	243 patients: -Fast-track group (*n* = 97, mean age 62 ± 14, 49 females); −Non-fast-track group (*n* = 146, mean age 59 ± 13, 66 females)	Yes	ACDF, PCDF, lumbar decompression, posterior lumbar fusion, lumbar microdiscectomy	1, 2, 3, 4, 5+	NR	NR	51 ± 30 h	3 incidental durotomy, 2 surgical site infection	1 instrumentation misplacement requiring operative revision, 1 disc herniation requiring reoperation	30 days	↓Opioid use at day 1 (26 ± 33 vs. 42 ± 409 MMEs) and in opiate-naive patients (16 ± 21 vs. 38 ± 36 MMEs), LOS (51 ± 30 vs. 62 ± 49 h) in fast-track group vs. non-fast-track group. =complications, 30 days readmission, 30 days reoperation time operation
Band et al. 2022 [64]	Prospective	32 patients: -Fast-track group (n = 16); −Non-fast-track group (*n* = 16)	Yes	Single-level MIS-TLIF for degenerative disease	NR	diabetes, hypertension	NR	1.6 days	NR	NR	NR	↓LOS (1.6 vs. 2.4 days), opioid consumption (51 mg MME vs. 320 mg MME) in fast-track group vs. non-fast-track group
Chen et al. 2022 [65]	Retrospective	207 patients: -Fast-track group (*n* = 112, mean age 52.86 ± 11.55, 49% females); −Non-fast-track group (n = 95, mean age 54.77 ± 11.66, 60% females)	Yes	Short-level (1- or 2-level) primary open PLIF for lumbar disc herniation, lumbar stenosis, and spondylolisthesis	NR	NR	NR	10.44 ± 3.07	Urinary retention (3.57%), constipation (10.71%), nausea and vomiting (5.36%), wound infection, venous thrombosis, fever, urinary tract infection, paravertebral hematoma, delirium	30-day readmission rate (4.46%), 30-day reoperation rate (2.68%)	30 days	↓LOS (10.44 ± 3.07 vs. 15.29 ± 3.57 days), off-bed time (7.53 ± 2.80 vs. 13.82 ± 3.44 days), complications (28.57% vs. 42.11%), urinary retention (3.57% vs. 11.58%), constipation (10.71% vs. 22.11%), nausea and vomiting (5.36% vs. 13.68%), drainage tube removal time (2 ± 0.65 vs. 3.53 ± 0.63), catheter removal time (1.79 ± 0.68 vs. 3.97 ± 1.15), surgical drainage at day 1–3 (165.20 ± 40.85 vs. 351.31 ± 32.49), intraoperative blood loss (126.61 ± 34.49 vs. 145.24 ± 22.52), financial cost (57,905.94 ± 12,463.50 vs. 62,683.68 ± 12,583.34 yuan), opioid consumption (18.98 ± 11.40 vs. 36.89 ± 15.30 mg), VAS score at day 3 (2.67 ± 1.02,vs. 3.51 ± 0.88), ODI score at day 3 (37.43 ± 10.22 vs. 41.19 ± 8.29), and ↑satisfaction (89.29% vs. 77.89%) in fast-track group vs. non-fast-track group. =wound infection, venous thrombosis, fever, urinary tract infection, paravertebral hematoma, delirium, operative time, 30-day readmission rate, 30-day reoperation rate
Leng et al. 2022 [66]	Retrospective	143 patients: -Fast-track group (*n* = 70, mean age 53.2 ± 9.3, 44% females); −Non-fast-track group (*n* = 73, mean age 52.07 ± 10.6, 61% females)	Yes	ACDF for cervical spondylosis, spondylotic myelopathy and radiculopathy	≥3	Diabetes mellitus, hypertension, chronic cardiovascular disease	NR	4 days	2.9% prolonged dysphagia, 1.4% hardware failure, 8.6% dysphagia/dysphonia, 1.4% nausea and vomiting	No 90-day readmission and reoperation	90 days	↓LOS (4 vs. 5 days), operative time, surgical drainage at day 1, costs, complications (dysphagia/dysphonia, hardware failure, nausea and vomiting), and ↑satisfaction, BMD in fast-track group vs. non-fast-track group. =prolonged dysphagia
Porche et al. b2022 [67]	Retrospective	114 patients: -Fast-track group (n = 57, mean age 66.1 ± 11.7, 53% females); −Non-fast-track group (*n* = 57, mean age 63.4 ± 13.3, 49% females)	Yes	1- or 2-level open TLIF for degenerative disease (spondylolisthesis, spinal stenosis, nerve root compression, recurrent disc herniation, pseudoarthrosis, or adjacent segment disease)	NR	NR	NR	3.6 ± 1.6 days	NR	2 reoperation within 30 days (3.5%, 1 hardware failure and 1 wound dehiscence)	30 days	↓Operative time (141 ± 37 vs. 170 ± 44 min), LOS (3.6 ± 1.6 vs. 4.6 ± 1.7 days), opioid consumption (8 ± 9 vs. 36 ± 38 MME), drains placed (40.4% vs. 96.5%), catheters placed (21% vs. 61%), PCA use (1.8% vs. 86%), and ↑first day of ambulation (0.6 vs. 1.3 days), bowel movement (2.2 vs. 3.0), bladder voiding (0.3 vs. 1.1 days) in fast-track group vs. non-fast-track group. =pain, EBL, complications, readmission rate, drain removal
Porche et al. a2022 [68]	Retrospective	58 patients: -Fast-track group (n = 17 frail, mean age 72.5 ± 4.2, 47% females; n = 26 non-frail, mean age, 73.4 ± 4.6, 62% females); −Non-fast-track group (n = 15 frail, mean age 73.2 ± 4.7, 20% females)	Yes	1- or 2-level open TLIF for spondylolisthesis, spinal stenosis, nerve root compression,recurrent disc herniation, pseudoarthrosis, or adjacent segment disease	NR	NR	NR	3.8 ± 1.9 days	NR	1 reoperation within 30 days with readmission (5.9%, instrumentation failure/wound dehiscence)	30 days	↑Physiological function (3.4 vs. 6.7 days), the first day of assisted-walking (0.7 vs. 1.6 days), first bowel movement (2.3 vs. 3.0 days), first day of bladder voiding (0.3 vs. 2.1 days) and ↓LOS (3.8 ± 1.9 vs. 4.8 ± 1.6 days), drains placed (59% vs. 100%), catheters placed (18% vs. 60%), PCA use (0% vs. 80%) in fast-track group vs. non-fast-track group. =opioid consumption, pain scores, operative time, EBL, complications, drai removal
Sun et al. 2022 [69]	Retrospective	166 patients: -Fast-track group (n = 86, mean age 56.919 ± 11.699, 69% females); −Non-fast-track group (*n* = 80, mean age, 58.863 ± 10.880, 56% females)	Yes	Lumbar fusion and internal fixation for lumbar spinal stenosis, spondylolisthesis or lumbar disk herniation	NR	NR	NR	10.465 ± 2.237 days	5.81% (3 delayed wound healing, 1 poor wound healing, 1 urinary system infection)	NR	NR	↓LOS (10.465 ± 2.237 vs. 12.050 ± 3.467 days), complication (5.81% vs. 16.25%), ODI score (25.276 ± 50.841 vs. 78.219 ± 3.540), cost (3.547 ± 0.746 vs. 3.746 ± 0.712 (ten thousand yuan), and ↑BI score (81.047 ± 24.479 vs. 21.400 ± 11.208), self-care ability of patients, dependent degree of patients in fast-track group vs. non-fast-track group. =operative time, EBL, VAS score
Wang et al. 2022 [70]	Retrospective	154 patients: -Fast-track group (*n* = 72, mean age 76.68 ± 4.83, 57% females); −Non-fast-track group (n = 82, mean age 76.38 ± 4.48, 68% females)	Yes	Long-level lumbar fusion for lumbar disc herniation or lumbar spinal stenosis	≥3	Renal, liver, connective tissue, cerebrovascular, peripheral vascular disease, diabetes, myocardial infarction	NR	17.74 ± 5.56 days	N = 6 (2 hypoproteinemia, 1 heart disease, 1 pneumonia, 1 urinary tract infection, 1 spinal fluid leakage)	*N* = 0	30 days	↓Complication (6 vs. 19), LOS (17.74 ± 5.56 vs. 22.13 ± 12.21 days) in fast-track group vs. non-fast-track group. =operative time, EBL, transfusion, VAS and ODI scores, readmission and mortality rates at 30-day
Zhang et al. 2022 [71]	Retrospective	119 patients: -Fast-track group (n = 56, mean age 52.94 ± 9.23, 39% females); −Non-fast-track group (*n* = 63, mean age 54.12 ± 10.34, 38% females)	Yes	Dynamic stabilization and discectomy for lumbar disk herniation (spinal stenosis secondary to disk herniation at 2 levels or less and disk herniation at 2 levels or less combined with intervertebral instability)	NR	NR	NR	7.12 ± 4.62 days	N = 0	N = 0	NR	↓VAS and ODI scores, EBL (90.52 ± 35.21 vs. 150.01 ± 70.34 ml), operative time (2.55 ± 1.35 vs. 3.25 ± 1.01 h), LOS (7.12 ± 4.62 vs. 9.66 ± 6.22 days), ambulation time (30.62 ± 17.68 vs. 48.22 ± 12.66 h), and ↑JOA score in fast-track group vs. non-fast-track group

*Abbreviations:Ref* references, *LOS* hospital length of stay, *ICU* intensive care unit, *EBL* estimated blood loss, ↓ decrease, vs. versus, ↑ increase, *n* number, *ER* emergency room, *IV* intravenous, *NR* not reported, *PLIF* posterior lumbar interbody fusion, *MIS* minimally invasive surgery, *CPAP/BiPAP* continuous positive airway pressure/bilevel positive airway pressure, *Hb* hemoglobin, *TLIF* transforaminal lumbar interbody fusion, *ODI* Oswestry Disability Index, *BPM-2* bone morphogenetic protein, *OLIF* oblique lumbar interbody fusion, *VAS* visual analog scale, *CRP* C-reaction protein, *JOA* Japanese Orthopaedic Association Score, *ALIF* anterior lumbar interbody fusion, *A&E* Accident and Emergency, *ACDF* anterior cervical discectomy and fusion, *PEEK* polyetheretherketone, *AIS* adolescent idiopathic scoliosis, *PSF* Posterior spinal fusion, *h* hours, *PETD* Percutaneous endoscopic transforaminal discectomy, *LDH* lumbar disc herniation, *NMS* neuromuscolar scoliosis, *PELIF* Percutaneous endoscopic lumbar interbody fusion, *TXA* tranexamic acid, *PIF* posterior instrumented fusion, *DVT* deep venous thrombosis, *PONV* postoperative nausea and vomiting, *MME* milligram morphine equivalents, *TL* Turkish Liras, *ACF* anterior cervical fusion, *CDA* cervical disc arthroplasty, *OFA* Opioid-free anesthesia, *OCA* opioid-containing anesthesia, *OMEs* oral morphine equivalents, *RCT* randomized controlled trial, *QoR40* Quality of Recovery 40, *DDD* degenerative disc disease,.*PROMs* Patient-Reported Outcome Measures, *NSAIDs* nonsteroidal anti-inflammatory drugs, *min* minutes, *PCDF* Posterior cervical decompression fusion, *BI* Barthel index

### Selection process

After submitted the articles to a public reference manager (Mendeley Desktop 1.19.8) to eliminate duplicates, possible relevant articles were screened using title and abstract by two reviewers (DC and FS). Studies that did not meet the inclusion criteria were excluded from review and any disagreement was resolved through discussion until a consensus was reached, or with the involvement of a third reviewer (MF). Subsequently, the remaining studies were included in the final stage of data extraction.

### Data collection process and synthesis methods

The data extraction and synthesis process started with cataloguing the studies detail. To increase validity and avoid omitting potentially findings for the synthesis, two authors (DC and FS) extracted and performed a Table (Table 1) taking into consideration: study design, patients’ number, age and gender, comparative analysis presence, surgery (indication and operation types), spine levels, comorbidities, intensive care unit length of stay (ICU LOS), hospital length of stay (LOS), complications, readmission and reoperation rates, follow-up, and outcomes/endpoints. The other Table (Table 2) takes into consideration fast-track procedures (pre-, intra, and postoperative). Preoperative components included patient education, consultation, physical therapy, nutrition and pain management. Intraoperative components included the day of surgery, anesthesia and pain management, fluid and blood transfusion, and nausea-vomiting prophylaxis. Finally, postoperative components included early mobilization, pain regimen, deep venous thrombosis (DVT) prophylaxis, nutrition status, early drain/catheter removal, antibiotic prophylaxis, fluid maintenance, and discharge.

**Table 2:** Pre-, intra- and postoperative fast-track procedures

Ref.	Preoperative				Intraoperative			
	Patient education/ consultation	Physical therapy	Nutrition	Pain menagement	Pre-op day	Anesthesia/ pain menagement	Fluid and blood transfusion	Nausea-vomiting prophylaxis
Adeyemo et al. 2021b [16]	Behavioral health, no smocking. Psychology, nutrition, mineral metabolism, geriatrics (> 65 yr) consultation	Yes	Yes	NR	NR	Anesthesia, epidural controlled analgesia	TXA, normotension, transfusion protocol (fresh frozen plasma after each 3 units PRBC, Hb = 10 g/dL, platelets< 100,000/μl after every 5 units of PRBC, cryoprecipitate for fibrinogen< 100 mg/dL)	NR
Adeyemo et al. 2021a [15]	NR	NR	NR	NR	NR	General anesthesia (endotracheal intubation), epidural patient-controlled analgesia (fentanyl 2 mg/mL, bupivacaine 0.625 mg/mL or 1.25 mg/mL at 6-8 mL/h, up to 2 mL boluses with lockout time of 15 min)	TXA, hemodynamic monitoring, cell saver blood salvage, fluid recovery (crystalloid, albumin, blood products)	NR
Angus et al. 2019 [17]	General informations, no smoking. Multidisciplinary consultation, vitamin D control, visits to reduce anxiety	Therapy prehabilitation	NR	NR	Carbohydrate load	Anesthesia, analgesia (lignocaine infusions, pre-incision ketamine boluses)	NR	NR
Brusko et al. 2019 [18]	NR	NR	NR	NR	NR	20 mL liposomal bupivacaine injection and 20 mL bupivacaine hydrochloride	NR	NR
Carr et al. 2019 [19]	General informations	NR	Nutrition and carbohydrate loading (300 ml clear, 2 h prior to hospital arrival and night before)	Multimodal analgesia (1 g acetaminophen the night before, 1.2 g gabapentin)	Multimodal analgesia (1 g acetaminophen the morning of surgery), active warming (prior to operating room), nasal povidone-iodine swab application	Total IV anesthesia (propofol, remifentanil to maintain), multimodal analgesia (0.5 mg/kg bolus ketamine and 0.5 mg/kg/h, IV acetaminophen at 6 h)	Normothermia (active warming with IV fluid warmers, covers, room temperature elevated prior to draping), fluid management (pulse pressure, stroke volume, cardiac output), TXA (1 g bolus prior to incision and 1 g over 8 h)	4 mg IV ondansetron
Chang et al. 2020 [20]	NR	NR	High protein diet, carbohydrate load	No narcotic medications	NR	IV anesthesia (propofol, ketamine, precedex, oxygen), multimodal analgesia (5-10 mL of 1:1 long-acting liposomal bupivacaine and 0.25% bupivacaine hydrochloride), no narcotic medications	NR	NR
Chen et al. 2021 [21]	General informations. Immunological tests, blood biochemistry, and coagulation, urine and stool examinations, RX, CT, MRI	NR	Fasting	NR	Antibiotics	Standard anesthetic protocol	Drain	NR
Dagal et al. 2019 [22]	General informations	NR	Nutritional support, carbohydrate loading	NR	NR	Anesthesia	GDHM, blood loss control (PPV, SVV, or CO), antifibrinolytics	NR
d’Astorg et al. 2020 [23]	General informations. Multidisciplinary consultation	NR	NR	NR	Hospitalization	Anesthesia with multimodal analgesia (dexamethasone, ketamine, few morphine derivatives, local anaesthetics)	Smallest number of catheters and drains	Antiemetics
Debono et al. 2019 [24]	General informations. Multidisciplinary consultation	NR	Modern fasting (until 6 h prior to surgery, clear liquids up to 2 h before, carbohydrate supplementation)	Limited premedication	Hospitalization, anti-infection prophylaxis	Short-acting anesthetics, pre-emptive analgesia	Use of drains limited	NR
Debono et al. 2021 [25]	General informations. Multidisciplinary consultation	NR	Modern fasting	Taken limited medication	Hospitalization, anti-infection prophylaxis (disinfection protocol)	Pre-emptive analgesia	No drain	NR
DeVries et al. 2020 [26]	General informations	NR	NR	NR	NR	Intrathecal morphine	NR	NR
Duojun et al. 2021 [27]	Oral and written education. Psychological consultation	NR	Diet (no water deprivation), prevention of gastrointestinal reactions (serotonin receptor antagonists)	NR	Skin preparation, 0.07–0.08 mg/kg midazolam (1 h before, 0.05–0.06 mg/kg in patients > 60 yr), oral 400 mg celecoxib (200 mg in patients > 70 yr or with BMI < 25 kg/m2)	Local anesthesia (1% lidocaine with maximum amount of 300 mg, 4 mg IV ondansetron hydrochloride), analgesia (40 mg IV parecoxib sodium, local subcutaneous injection of ropivacaine)	Normothermia (36 °C, insulation blanket, heating fan), vascular condition control	4 mg IV ondansetron hydrochloride
Feng et al. 2019 [28]	General informations (handout)	NR	Fasting carbohydrate loading (6 h for liquids, 8 h for solid food and short-chain polypeptides drinks, 2 h for clear liquids)	NR	Pre-emptive analgesia (oral celecoxib 200 mg and pregabalin 150 mg 1 h before), antimicrobial prophylaxis (1.5 g cefuroxime 1 h before)	LIA (ropivacaine), catheters	TXA, normovolemia (goal-directed fluid administration), normothermia (> 36 °C, convective warming device)	NR
Flanders et al. 2020 [29]	Written general information. Nutritional consultation (BMI < 18.5 or > 25 kg/m2 or with serum albumin < 3.5 g/dL), pain management (> 30 morphine equivalents of opioids for > 4 weeks), sleep medicine (scoring > 2 on the STOP-BANG questionnaire), endocrinology for clearance (serum glucose > 200 g/dL or HbA1c > 8%)	NR	Carbohydrate load (Gatorade, day before surgery and 2 h before arriving at hospital)	NR	NR	Multimodal analgesia (gabapentin with opioid and nonopioid analgesics)	NR	NR
Fletcher et al. 2020 [30]	Pulmonology, gastroenterology, neurology and other specialists’ consultation	NR	NR	NR	NR	NR	NR	NR
Fletcher et al. 2021 [31]	NR	NR	NR	NR	NR	NR	NR	NR
Garg et al. 2021 [32]	General informations, no smoking and alcohol. Preanesthetic (HbA1c < 7 for diabetic patients) and nutritional (if BMI < 18.5 or > 30, hematinics for anaemia, protein supplementation for poorly nourished patients) consultation; bone mineral density evaluation (calcium and vitamin D supplementation, teriparatide injection, for osteoporotic patients)	Rehabilitation	Fasting (6 h before for solid food, 2 h before for clear liquids, IV 5% dextrose solution 500-1000 ml, overnight before surgery)	NR	Preemptive analgesia (75 mg oral pregabalin, 1000 mg acetaminophen, 2 h before surgery), chlorhexidine (4% the night before and morning of surgery), nasal swab (5 days before to detect *Staphylococcus aureus*, 2% nasal mupirocin, vancomycin)	Multimodal total IV anesthesia with < 0.5% MAC, avoidance of IV long-acting opioids, infiltration of subfascial local anesthetic drugs (0.25% bupivacaine before wound closure), antibiotic prophylaxis (cefuroxime injection), 2 to 3 of 3.5% povidone-iodine infused pulsatile lavage for wound	TXA bolus (20 mg/kg) and infusion (2 mg/kg/h), warmed IV fluids, invasive blood pressure monitoring, normothermia maintenance (> 36°, convection warmers)	8 mg dexamethasone, 4 mg ondansetron (30 to 45 min before emergence from anesthesia)
Gong et al. 2021 [33]	General informations. Nutritional (protein, glucose, omega-3 fatty acids, and specific amino acids, glutamine, arginine) consultation	NR	Fasting (4 h for liquids, 6 h for solid), carbohydrate loading (clear carbohydrate-rich drink 4 h before surgery)	Pre-emptive oral analgesics (celecoxib, eperisone, extended-release tramadol, pregabalin, on day of admission)	Antimicrobial prophylaxis (1.5 g cefuroxime 30 min before incision)	Surgical wound local anesthetic (skin blocks around the skin incision)	Normothermia (> 36 °C, air-warming device and warmed IV fluids), normovolemia (goal-directed fluid therapy)	NR
He et al. 2020 [34]	NR	NR	NR	NR	TXA IV bolus 10 mg/kg (15 min before skin incision)	General anesthesia, TXA IV infusion 6-8 mg/kg/h (up to 15 mg/kg), drains	NR	NR
Heo et al. 2019 [35]	General informations. Emotional support	NR	NR	NR	Pre-emptive analgesic (pregabalin 75 mg or gabapentin 300 mg), prophylactic antibiotic injection (first-generation cephalosporin), IV TXA, IV antiemetics	General or epidural anesthesia, local anesthetic injection, IV secondary prophylactic antibiotic injection, drainage catheter (epidural hematoma prevention), vancomycin local infiltration (over wound areas)	Maintain IV TXA	NR
Ifrach et al. 2020 [36]	General informations and no smoking. Pain management (> 30 MED of opioids for > 4 weeks), sleep medicine (scoring > 2 on STOP-BANG questionnaire), endocrine clearance (serum glucose > 200 g/dl or HbA1c > 8%), nutritional consultation (BMI < 18.5 or > 25 kg/m2 or with serum albumin level < 3.5 g/dL)	NR	Carbohydrate load (gatorade, day before surgery and 2 h before arriving at hospital)	NR	NR	Anesthesia, multimodal pain therapy (gabapentin 600 mg at day 0), catheters limited	NR	NR
Jazini et al. 2021 [37]	General informations and no smoking. Medical, cardiology, nutritional, pain management physicians consultations	NR	Yes	600 mg gabapentin, 1000 mg acetaminophen, 200 mg celecoxib, 750 mg methocarbamol, 15 mg extended-release morphine	Carbohydrate rich drinks the night before surgery and 4 h prior to surgery, clear liquids until 2 h prior to surgery	Anesthesia monitored (transversus abdominis plane blocks) or TIVA if necessary, antibiotics, local anesthetics, 0.25% bupivacaine with epinephrine (into local subcutaneous and intramuscular tissues), long-acting opioids, opioid patient-controlled analgesia, IV opioid analgesia for breakthrough pain	Normothermia (35 °C), 2 g IV magnesium bolus, 10 mg/kg TXA bolus (EBL > 200 cc), IV lidocaine and ketamine drips, normoeuvolemia (hemodynamic monitoring, goal-directed fluids, lactated ringers)	4 mg ondansetron, scopolamine patch
Julien-Marsollier et al. 2020 [38]	General informations	NR	Fasting minimization, systematic iron supplementation (if hemoglobin < 14 g dl-1), recombinant erythropoietin	Oral 800 mg gabapentin	Clear-liquid carbohydrate loading (apple juice and water until 2 h prior to surgery), antibiotics	Anesthesia (dexmedetomidine and ketamine) and maintenance (sevoflurane in a 50% mixture of O_2_/N_2_O, bispectral index values 40–60), nonopioid analgesia (30 min before the end of surgery, IV or oral paracetamol 15 mg kg-1 6 h, IV ketoprofen 1 mg kg − 1 8 h or oral ibuprofen 10 mg kg − 1 6 h, nefopam 0.25 mg kg − 1 6 h, dexamethasone 0.15 mg kg − 1, 5μgkg − 1 intrathecal morphine)	TXA 10 mgkg − 1 and continuous infusion of 5mgkg − 1 h − 1, muscle relaxant, maintenance IV fluid (Ringer’s lactate), normothermia (36.5°-37 °C, double warmer system), sufentanil boluses (arterial pressure and heart rate within 20% of preoperative values), transfusion target Hb = 8gdL − 1	Ondansetron 0.1 mg kg − 1 8 h
Kalinin et al. 2021 [39]	General informations and no smoking. Anesthesiologist consultation	NR	Fasting	Avoid premedication	Solid food 6 h before surgery, fluids 2 h before, antibiotic prophylaxis (2 h before the first incision)	Dexmedetomidine (to control depth of anesthesia), sugammadex (for fast and effective reversal of the neuromuscular block upon patient extubation), local anesthetics infiltration (around surgical wound before suturing), multimodal analgesia (NSAIDs prior to skin incision and suturing)	NR	Compression hosiery, ultrasound examination of lower limb veins (before and next day after surgery)
Kerolus et al. 2021 [40]	General informations	NR	Fasting	Pre-anesthetic medication, pregabalin 100 mg, oxycodone extended release 10 mg (> 75 yr old), baclofen 10 mg	NR	General anesthesia (ketamine, propofol or inhaled anesthetics as isoflurane or sevoflurane), IV opioids (fentanyl and its derivatives minimized), paralytics (if necessary), IV acetaminophen 1000 mg, local anesthetic 5–15-20 cc (0.25% ropivacaine with 1:100000 epinephrine, subcutaneously prior to closure), minimize drains	NR	4 mg ondansetron, if necessary, every 6 h, 10 mg metoclopramide, if necessary, every 6 h
Kilic et al. 2019 [41]	General informations. Anesthesiologists, surgeons, nurses, psychological consultation	NR	Fasting	NR	Antibiotic prophylaxis (30 min before incision), clear fluids up to 2 h and solid foods up to 4 h before surgery	TIVA (fentanyl 1 mg/kg and 2 mg/kg propofol), oxygen ventilation (endotracheal tube), anesthesia maintain (IV 2–4 mg/kg/hr. propofol), analgesia (30-ml bolus with 0.5% bupivacaine hydrochloride into subcutaneous tissue after closure, IV acetaminophen 1000 mg), no nasogastric tubes or catheter or drains	Fluid management restricted, systolic blood pressure, diastolic blood pressure, heart rate, and peripheral oxygen saturation monitoring (before and after anesthesia), normothermia (36 °C, convective warming devices), euvolemia (500 ml fluids), vasopressors (in case of hypotension)	IV 0.15 mg/kg ondansetron and 0.2 mg/kg dexamethasone
Kilic et al. 2020 [42]	General informations and no smoking	Preconditioning exercises	NR	Upon arrival analgesia (oral gabapent 300 mg and acetaminophen 1000 mg), no opioids	Admission (same day of surgery), antibiotic prophylaxis (30 min before incision), clear fluids 2 h and solid food 4 h before surgery	TIVA (bispectral index monitoring and hypotensive anesthesia maintain), no opioid, 30 cc marcaine hydrochloride 0.5% into the subcutaneous tissues after wound closure, no nasogastric tubes or catheters or drains, ICU admissions minimized	IV 1.5 g TXA and topically 1 g in 100 mL saline during suturing and at the end of operation, fluid management and blood transfusions restricted (blood products minimized and transfusion if Hb < 8 g/dL), normothermia (convective warming devices)	IV 0.15 mg/kg ondansetron, 0.2 mg/kg dexamethasone
Kim et al. 2021 [43]	General informations. Screening program, chronic pain service consultation	NR	NR	NR	NR	Anesthesia	Blood loss minimize (< 300-400 cc equivalent to 125 cc of cell saver blood return, 10 mg/kg TXA before incision and 1 mg/kg until closure, arterial pressures < 65, short-acting paralytic, local hemostatic agents collagen- and thrombin-based), transfusion minimize (EBL kept at 20% or less of the total blood volume)	NR
Lampilas et al. 2021 [44]	General informations. Nurses and anesthetist consultation	Physiotherapy	Improved fasting and energy drink	NR	Hospitalization (1.30 h before surgery), energy drink (2 h before surgery)	Analgesic wound infiltration (2 mg/kg before incision), analgesic (remifentanil, ketamine, 0.15 mg/kg bolus morphin 1 h before end of surgery), hypnotic drugs (propofol, desflurane), catheter and drain avoided	TXA, hypothermia prevention	Dexamethasone, droperidol, zophren, if necessary
Li et al. 2018 [45]	General informations on pain coping, discharge criteria andfollow-up informations	NR	No bowel preparation	NR	Fasting 6 h and water 2 h before, antimicrobial prophylaxis	Local anesthesia (0.75% ropivacaine), multimodal analgesia (IV NSAIDs, 40 mg parecoxib every 12 h or 100 mg flurbiprofen for 3 days and oral 100 mg celecoxib)	Operation room (25 °C) and body temperature maintenance (warm fluids air-warming devices)	5-HT receptor antagonist
Li et al. 2020 [46]	Verbal and handouts general informations. Nutritional consultation	NR	Fasting	NR	Clear fluids and carbohydrate drink up to 2 h before surgery, antimicrobial prophylaxis (within 1 h of incision)	TIVA (propofol, lidocaine, ketamine, ketorolac, antiemetics, up to 0.5% MAC inhaled anesthetics), LIA, multimodal analgesia	TXA, normothermia (36–37 °C), euvolemia (salt and water overload avoidance)	NR
Li et al. 2021 [47]	Verbal and handouts general informations. Nutritional consultations	NR	Fasting	NR	Clear fluids and carbohydrate drink up to 2 h before surgery, antimicrobial prophylaxis (within 1 h of incision)	TIVA (propofol, lidocaine, ketamine, ketorolac, antiemetics, up to 0.5% MAC inhaled anesthetics), LIA, multimodal analgesia	TXA, normothermia (36–37 °C), euvolemia (salt and water overload avoidance)	NR
Nazarenko et al. 2016 [48]	General informations. Neurosurgeon, anesthesiologist consultations	NR	NR	NR	Hospedalization	Regional anesthesia	NR	NR
Rao et al. 2021 [49]	Education booklet	NR	Iron supplementation, multivitamin, bowel regimen (senna, 24 h before)	NR	Hospitalization, scopolamine patch, pregabalin or liquid gabapentin, antibiotic prophylaxis, cleansing (chlorhexidine)	Aminocaproic acid (bolus 100 mg/kg and infusion 10 mg/kg/h), epidural catheter, dexamethasone (prior to incision), IV acetaminophen (during closure)	Air warming blanket, IV fluid warmers	Ondansetron (prior to emergence for antiemesis)
Shaw et al. 2021 [50]	NR	NR	NR	NR	NR	Methadone (29.5 MME, 0.5 MME/kg or 0.1 mg/kg)	NR	NR
Smith et al. 2019 [51]	Education packet, antibiotics prophylaxis (ancef 2 g or 3 g if > 120 kg, clindamycin 900 mg, or vancomycin 15 mg/kg)	Yes	NR	NR	NR	Anesthesia, multimodal analgesia (acetaminophen 975 mg, gabapentin 900 mg, ketamine 30 mg IV for patients with >risk for pain), dexamethasone 8 mg IV after induction of anesthesia, antibiotics (1 h prior to incision), fentanyl, morphine, or hydromorphone, patient-controlled analgesia, methocarbamol 1500 mg IV	NR	Ondansetron 4 mg IV, oral aprepitant 40 mg for high-risk patients
Soffin et al. 2019b [52]	General information. Multidisciplinary consultations	NR	NR	NR	Fasting (4 h for liquid, 6 h for solid), carbohydrate loading (12.5% maltodextrin-based drink 4 h before surgery), oral pre-emptive analgesia (oral 1000 mg acetaminophen and 300 mg gabapentin within 60 min of surgery), antimicrobial prophylaxis (within 1 h of incision)	TIVA with up to 0.5% MAC inhaled anesthetics (50-100 mg/kg/min propofol and 0.1–0.5 mg/min ketamine), non-opioid analgesia (15–30 mg ketorolac, 1–2 mg/kg/h lidocaine, LIA, ossicodone if necessary), no drain/catheter	Normothermia (convective warming, 36 °C), normovolemia (IV fluids)	1.5 mg scopolamine transdermal, IV 4-8 mg ondansetron 30 min before, 4-8 mg dexamethasone
Soffin et al. 2019a [53]	General informations	NR	Nutrition	NR	Solids until 6 h, clear liquids until 4 h prior to surgery, carbohydrate loading (4 h prior to surgery), antibiotic prophylaxis within 60 min of incision, pre-emptive analgesia (oral 1000 mg acetaminophen, 300 mg gabapentin)	TIVA (propofol 50–100 μg∙kg∙min − 1 and ketamine 0.1–0.5 mg∙min − 1, up to 0.5 MAC as needed, but avoid N2O), multimodal analgesia (lidocaine bolus 1 mg∙kg − 1 on induction and infusion 2 mg∙kg − 1 until closure of incision, ketorolac 15-30 mg during closure, IV acetaminophen 1000 mg), topical methylprednisolone prior to closure, no drain and catheter	Normothermia (36.0–37.0 °C, convective warmers), normovolemia (IV fluid warmer restriction 10–15 ml∙kg − 1), arterial pressure maintenance within 20% of baseline with ephedrine 5–10 mg IV doses as needed	1.5 mg transdermal scopolamine, dexamethasone 4-8 mg, ondansetron 4 mg
Soffin et al. 2019c [54]	General informations	NR	NR	NR	Oral 1000 mg acetaminophen and 300 mg gabapentin	General anesthesia (endotracheal intubation), premedication (midazolam 0.05 mg/kg), anesthetic induction (100% oxygen, 10 L/min, propofol 1.5–2 mg/kg, lidocaine 1.5 mg/kg, vecuronium 0.1 mg/kg, fentanyl 2 μg/kg), boluses fentanyl 1–2 μg/kg, or dilaudid up to 2 mg, propofol 50–150 μg/kg/h, ketamine (0.1–0.5 mg/min), lidocaine (2 mg/kg/h until closure of incision), isoflurane or sevoflurane up to 0.5 MAC as needed, mechanical ventilation (1:1 mixture of oxygen:air FiO2 50%, tidal volume 6–8 ml/kg, respiratory rate 8–14 titrated to an end-tidal carbon dioxide between 30 and 35 mmHg), residual neuromuscular blockade reversed with glycopyrrolate and neostigmine, IV labetalol 10 mg and/or propofol up to 50-mg bolus, and/or increased MAC inhaled anesthetics (if arterial pressure > 100 and/or heart rate 15% above baseline), opioids (fentanyl 1–2 μg/kg or dilaudid up to 2 mg if necessary)	Electrocardiography, blood pressure monitor, pulse oximetry, IV crystalloid solution (lactated Ringer’s solution 8–12 ml/kg/hr), arterial pressure within ±20% of each patient’s baseline value	IV 10 mg metoclopramide or ondansetron 4 mg, scopolamine 1.5 mg transdermally if refractory PONV, 4-8 mg dexamethasone, 4 mg ondansetron, 15-30 mg ketorolac during closure
Soffin et al. 2020 [55]	Education module	NR	NR	NR	Fasting and 125-ml clear carbohydrate-rich beverage (4 h before), pre-emptive analgesia (oral 300 mg gabapentin, 1000 mg acetaminophen within 60 min), antimicrobial prophylaxis (within 1 h)	IV anesthesia (1–2 mg·kg-1 and 25-100 μg·kg·min-1 propofol, 0.1 mg·kg-1 vecuronium, up to 2 μg·kg-1 fentanyl, 0.1–0.5 mg·min-1 ketamine, 0.3–0.5 μg·kg·h-1 dexmedetomidine, infusions with isoflurane in oxygen-enriched air up to 0.3 minimum alveolar concentration), multimodal analgesia (IV 15-30 mg ketorolac, 1 mg·kg-1 lidocaine bolus and 2 mg·kg·h-1 infusion)	Normothermia (36–38°, forced-air warming blanket), normovolemia (warmed IV fluid)	Scopolamine patches (if high risk), 4-8 mg dexamethasone, 4 mg ondansetron
Staartjes et al. 2019 [56]	General informations. Anesthesiologic screening, cardiologist, nutritional (BMI > 30 kg/m2) consultations	NR	NR	NR	Antimicrobial prophylaxis (broad-spectrum antibiotic), low-molecular-weight heparin	General anesthesia (propofol, sufentanil), LIA (2.5 mg/ml ropivacaine intramuscularly prior to incision), muscle relaxants limited	Hypothermia prevention (warm-air blankets), fluid imbalance and blood transfusion prevention, vasopressors, autologous cell-salvage	NR
Venkata et al. 2018 [57]	General informations. Physician and anesthesiological consultations	NR	NR	NR	NR	General anesthesia, LIA (20 ml 0.25% bupvicaine during or after closure), multimodal analgesia (remifentanil hydrochloride, IV paracetamol, COX-2 inhibitor paracoxib sodium, small dose of morphine), antibiotic prophylaxis (1.5 g cefuroxime at induction anesthesia, chlorhexidine skin cleanse of operative site), no drain or catheter	No transfusion	NR
Wang et al. 2017 [58]	General informations	NR	Enteral nutrition (protein uptake)	NR	Fasting (8 h for liquids, 12 h for solids), carbohydrate loading, antimicrobial prophylaxis and skin preparation (first-generation cephalosporin 1 h before incision, vaccination for MRSA)	Anesthesia (short-duration sedation, IV propofol and ketamine, oxygen), local analgesia (long-acting liposomal bupivacaine), osteobiologic adjuvants, no drains, catheter and narcotic medications	Normothermia and blood pressure maintain, fluid balance (cardiac output monitoring)	NR
Wang et al. 2020 [59]	General informations	NR	Fasting, fluid and carbohydrate loading	NR	Antimicrobial prophylaxis	Standard anesthetic protocol, LIA	TXA, normothermia maintein	NR
Yang et al. 2020a [60]	General informations	NR	Diet	NR	Clear liquid diet (day of surgery), neurontin (30 min before)	Intrathecal morphine (at the start of procedure)	NR	NR
Yang et al. 2020b [61]	General informations. Nutrition and psychological consultations	NR	Increased albumin infusion and improved enteral uptake	Preemptive analgesia (muscle relaxant, NSAID, celecoxib, meloxicam, flurbiprofen, or tramadol), opioid restricted, respiratory infection prevention (gentamicin, mucosolvan and albuterol aerosol, twice a day for 2 days before the start of endotracheal anesthesia), pre-anaesthesia (1 day before anesthesia initiation)	Fasting (2 h for liquids, 6 h for solid food), carbohydrate loading (water for cases with diabetes), preventive analgesia (IV NSAID, 5 min before anesthesia induction)	General anesthesia (fentanyl, short-acting remifentanil if necessary), LIA (long-acting liposomal ropivacaine before wound suturing)	Multiple monitoring (electrocardiogram, blood pressure, arterial blood gas analysis, bispectral index, stroke volume variation, urinary volume, oxygen saturation and end tidal CO2), crystalloid solution infusion (1 − 2 ml/kg h with or without colloidal fluid), normothermia (warm draping, infused fluid heated, > 36 °C)	NR
Yang et al. 2021 [62]	General informations. Cardiac and pulmonary function, nutritional status, mental health consultations	Preconditioning exercise (6 weeks before surgery), balloon blowing	NR	NR	Antibiotic prophylaxis (within 0.5-1 h of incision and additional antibiotic if operation time > 180 min), fasting (clear fluids up to 2 h and solids up to 6 h before anesthesia, carbohydrate-contained beverage or high-dose glucose infusion ≥5 mg/kg/min)	General anesthesia (IV propofol 1–2 mg/kg, midazolam 1–2 mg, sufentanil 0.3–0.6 mg/kg, rocuronium 0.6 mg/kg), multimodal analgesia IV (parecoxib 40 mg, oxycodone 0.1–0.2 mg/kg within 0.5 h of induction, remifentanil 0.1–0.3 mg/kg/min, dexmedetomidine 0.4 mg/kg/h, propofol 4-12 mg/kg/h, opioids, COX-2) inhibitor), subcutaneous drainage	Restricted fluid therapy, temperature management (36 °C, fluid warming, airway humidification, forced-air warming blanket), antipressure ulcers nursing (foam pads), blood management (hypotensive anesthesia with arterial pressure 70-75 mmHg, cell salvage, TXA 10-20 mg/kg before incision + 1 mg/kg/h infusion + 3 g topical application, blood products transfusion if Hb < 70 g/L)	Dual antiemetic prophylactic therapy with IV ondansetron 4 mg, dexamethasone 10 mg, or intramuscularly metoclopramide 10 mg
Young et al. 2021 [63]	General informations and no smoking. Chlorhexidine for skin, screening for diabetes mellitus, malnutrition and methicillin-*Staphylococcus aureus* colonization, neuropsychology clinic, multidisciplinary anesthesia pain management	Yes	Standard bowel regimen, diet	NR	Analgesia (1 g acetaminophen, 600-1200 mg gabapentin 60 min before surgery), infection prophylaxis (2 g cefazolin or clindamycin/vancomycin 30-60 min before incision)	Local anesthetic (bupivacaine, and epidural morphine sulfate in nonfusion cases), catheter	NR	10 mg dexamethasone
Band et al. 2022 [64]	Optimization of chronic disease management (diabetes, hypertension), discussion regarding weight loss, no smocking, preoperative education, counseling, and hospital orientation session	NR	NR	Oral analgesics (acetaminophen 1 g, baclofen 10 mg, oxycontin 20 mg, gabapentin 300 mg)	NR	General anesthesia (midazolam, ketamine 0.5 mg/kg bolus up to max of 50 mg followed by 0.5 mg/kg/h, and/or dexmedetomidine infusion 0.4mcg/kg/h), dexamethasone 10 mg), subfascial drains, peri-incisional bupivacaine 0.5% 20Ml, urinary catheters	NR	Zofran 4 mg
Chen et al. 2022 [65]	Education, assessment, diet management, smoking and alcohol cessation, psychological evaluation	NR	Fasting (6 h for liquids and 8 h for solid food), clear fluids, including carbohydrate drinks up to 2 h before	Oral celecoxib 200 mg and pregabalin 150 mg within 1 h before surgery	IV first-generation cephalosporin for 30 min before	Local anesthesia, long-acting opioids, anesthetic agents, and large doses of muscle relaxants avoided	Normothermia (heating device, 36 °C), goal-directed fluid administration, IV tranexamic acid (10-20 mg/kg load dose before resection followed by infusion at 1 mg/kg/h maintenance dose)	NR
Leng et al. 2022 [66]	Education, consultation, smoking cessation	NR	Modern fasting (solids within 6 h and carbohydrate beverages within 2 h prior)	Oral celecoxib 200 mg, pregabalin 75 mg, acetaminophen 1 g, 1 h before	Antimicrobial prophylaxis (cefuroxime 1.5 g, 30 min before)	Anesthesia, local analgesia (5 mg/mL ropivacaine hydrochloride), catheter	TXA (1 g bolus followed by 0.5 g/h infusion), dexamethasone 10 mg, normovolemia (goal-directed fuid administration, vasopressors), normothermia (36 °C, convective warming device)	5-HT receptor antagonist (ramosetron)
Porche et al. b2022 [67]	Physical capacity, cognitive, cardiac, pulmonary, renal, pain, nutrition and risk evaluation, anemia and diabetes control, education, medical optimization, smoking cessation	NR	Clear liquids for 2 h and solid foods for 8 h cessation before	NR	Acetaminophen, duloxetine 60 mg, gabapentin, methadone (0.1–0.2 mg/kg with max 20 mg), antifibrinolytics	Induction and maintenance and airway management, total IV anesthesia if applicable, adjuvant pain management (dexmedetomidine, ketamine, lidocaine), IV methadone, no NSAIDs or steroids	Goal-directed fluid therapy, baseline ABG and TEG, colloids for boluses per EBL and SVV, transfuse packed red blood cells if Hb < 8 or < 9 in coronary artery disease patients, transfuse platelets and cryoprecipitate per TEG, avoid fresh frozen plasma unless indicated, normothermia (warm fluids and upper and lower body air-warming devices)	NR
Porche et al. a2022 [68]	Physical capacity, cognitive, cardiac, pulmonary, renal, pain, nutrition and risk evaluation, anemia and diabetes control, education, medical optimization, smoking cessation	NR	Clear liquids for 2 h and solid foods for 8 h cessation before	NR	Acetaminophen, duloxetine 60 mg, gabapentin, methadone (0.1–0.2 mg/kg with max 20 mg), antifibrinolytics	Induction and maintenance and airway management, total IV anesthesia if applicable, adjuvant pain management (dexmedetomidine, ketamine, lidocaine), IV methadone, no NSAIDs or steroids	Goal-directed fluid therapy, baseline ABG and TEG, colloids for boluses per EBL and SVV, transfuse packed red blood cells if Hb < 8 or < 9 in coronary artery disease patients, transfuse platelets and cryoprecipitate per TEG, avoid fresh frozen plasma unless indicated, normothermia (warm fluids and upper and lower body air-warming devices)	NR
Sun et al. 2022 [69]	Education, management of nutrition, dietary, sleep, pain and body temperature	NR	NR	NR	NR	NR	Liquid therapy	NR
Wang et al. 2022 [70]	Patient education and counseling	NR	No prolonged fasting, eat up to 6 h before and carbohydrate drinks up to 2 h before	NR	Antibiotic prophylaxiswithin 1 h, TXA within half h	Multimodal analgesia, local infiltration analgesia (10 mL ropivacaine and 10 mL lidocaine)	Normothermia (convective warming, 36–37 °C)	NR
Zhang et al. 2022 [71]	Counselling, education	NR	Fasting and water deprivation for 2 h	Analgesic therapy (oral etoricoxib 120 mg the day before surgery)	TXA	Continuous epidural anesthesia, no catheter	TXA, nerve electrophysiological monitoring	NR

Ref.	Postoperative							
	Mobilization	Pain regimen	DVT prophylaxis	Nutrition	Early drain/catheter removal	Antibiotic prophylaxis	Fluid	Discharge
Adeyemo et al. 2021b [16]	Day 1	Multimodal analgesia, narcotic medication minimization	Compression stockings, low molecular weight heparin	Appropriate nutritional intake	NR	NR	NR	NR
Adeyemo et al. 2021a [15]	NR	Epidural patient-controlled analgesia with catheter removal on day 3 (muscle relaxants, gabapentin, paracetamol, narcotic)	NR	NR	NR	NR	NR	NR
Angus et al. 2019 [17]	Yes	Multimodal analgesia (patient-controlled analgesia opioids/ketamine and IV paracetamol)	Yes	Bowel regimen	Drain	Wound care	NR	Support line (a call at day 1 and 3 post discharge and clinic review at 6 days)
Brusko et al. 2019 [18]	Physical therapy, occupational therapy	1 g IV acetaminophen infusion, 5 mg–325 mg oxycodone-acetaminophen tablets	NR	NR	NR	NR	NR	Daily visits from the multidisciplinary team
Carr et al. 2019 [19]	Day 2	8 mg/h ketamine (for the first 24 h after surgery), 1 g acetaminophen and 900 mg gabapentin (for 3 days)	NR	Oral intake and full diet	Day 2 (catheter)	NR	Maintenance IV fluids (2 ml/kg/h)	NR
Chang et al. 2020 [20]	Yes	Standard and PRN opioid medications (percocet 5–325, tramadol, dilaudid IV)	NR	NR	NR	NR	NR	NR
Chen et al. 2021 [21]	Yes	Multimodal analgesia	Yes	Early feeding, gastrointestinal management	Within 48 h (catheter and drain)	Antibiotics	NR	Visits, blood and coagulation examinations, blood biochemistry, RX, CT, RM, discharged 3–5 days after surgery
Dagal et al. 2019 [22]	NR	Opioid-sparing multimodal analgesia (acetaminophen, gabapentin, ketamine)	NR	NR	NR	NR	NR	NR
d’Astorg et al. 2020 [23]	Day 0	Oral analgesia	NR	NR	Catheters and drains	NR	NR	Follow-up phone call (day 1), surgical consultation (4–6 weeks)
Debono et al. 2019 [24]	Physiotherapy	Opioid-sparing multimodal approach (tramadol, NSAIDs, and oxycodone if necessary)	NR	Early oral feeding	After surgery (catheter)	NR	NR	Rapid discharge, online/phone survey, mobile app (15 days), surgical consultation (6 weeks)
Debono et al. 2021 [25]	Physiotherapy	NR	NR	NR	NR	NR	NR	Day 1 (discharge), mobile app (15 days), surgical consultation (6 weeks), satisfaction phone survey, online clinical evaluation
DeVries et al. 2020 [26]	Day 2	Patient-controlled analgesia (acetaminophen, NSAID), oral opioids	NR	NR	Day 2 (catheter)	NR	NR	Day 3
Duojun et al. 2021 [27]	3 h after surgery rehabilitation exercise: lumbar back muscle function	Multimodal and advanced analgesia (active administration, avoiding opioid use, NSAIDs use)	NR	Early high-quality diet	NR	NR	No rehydration	Within 3 days
Feng et al. 2019 [28]	Day 1	Opioid sparing multimodal analgesia (IV parecoxib 40 mg, oral celecoxib 200 mg every 12 h, oral pregabalin 75 mg every 12 h, if necessary intramuscular tramadol 100 mg)	NR	Early nutrition (clear liquids day 0, oral intake day 1), oral diet	After surgery (catheters)	NR	NR	NR
Flanders et al. 2020 [29]	Within 6 h and ambulation 3 to 5 times daily (day 1)	Nonopioids, muscle relaxants, oral and IV opioids as needed	Active exercises	Nutrition, chewing gum and instructed to chew (1 piece 3 times per day to reduce the risk of ileus)	NR	Wound washing (for 2 weeks)	NR	NR
Fletcher et al. 2020 [30]	Yes	IV enteral narcotic and antispasmodies (diazepam)	NR	Normal feeding with clear liquids	Day 1 (catheters and drains)	NR	NR	NR
Fletcher et al. 2021 [31]	NR	Oral medication	NR	Regular diet	NR	NR	NR	Patient/family comfort with care plan
Garg et al. 2021 [32]	Yes	Opioid-sparing multimodal analgesia (acetaminophen, pregabalin, and diclofenac for breakthrough pain, limited use of NSAIDs, avoidance of tramadol)	NR	Early enteral feeding and chewing gum	Drain avoided (if applied, removed between 24 and 36 h after surgery), catheterization avoided (if applied, removed day 1)	NR	IV fluids discontinued (within 6 h of surgery)	Early discharge, telephonic follow-up (48 h and 1 week after discharge)
Gong et al. 2021 [33]	Day 1	Multimodal analgesia (IV parecoxib, oral celecoxib, acetaminophen, and if necessary, pregabalin from day 1, IV or intramuscular morphine if necessary)	NR	Early (liquid food day 0, solid food day 1)	Day 1 (catheter and drain), drain output < 20 mL/24 h	NR	NR	NR
He et al. 2020 [34]	NR	NR	Intermittent pneumatic compression device	NR	Drain (< 30 ml for 24 h)	NR	NR	NR
Heo et al. 2019 [35]	Yes	IV patient-controlled analgesia, oral analgesic w/ pregabalin or gabapentin	Ambulation, anti-DVT stocking, intermittent pneumatic compression of legs	Early nutrition	NR	NR	NR	NR
Ifrach et al. 2020 [36]	Within 6 h of surgery, ambulation (3–5 times daily ketorolac on day 1)	Pain management (acetaminophen 975 mg at day 0 every 6 h, IV ketorolac 15 mg as needed, muscle relaxants as cyclobenzaprine 10 mg and diazepam 5 mg as needed, oral opioid as oxycodone 5-10 mg or hydromorphone 2-4 mg at day 0 as needed, IV opioids as morphine 1–2 mg and hydromorphone 0.2–0.4 mg if necessary for breakthrough pain until day 1)	Sequential compression device, heparin every 8 h starting day 1	Nutrition, chewing gum (1 piece 3 times per day, Senna 17.2 mg twice a day, polyethylene glycol as needed, to reduce the risk of postoperative ileus)	NR	Standard open wound care regimen (daily chlorhexidine bath beginning day 1)	NR	Primary care within 2 weeks from discharge
Jazini et al. 2021 [37]	Day 0	Multimodal analgesia every 8 h (300 mg gabapentin, 1000 mg acetaminophen, 750 mg methocarbamol, 20 mg famotidine), 5 mg oxycodone (1–3/10 pain), 10 mg (4–6/10 pain), 15 mg (7–10/10 pain), 8 mg hydromorphone for breakthrough pain, incentive spirometry 10 times every h	NR	Advanced diet, protein shakes, stool softeners	Day 1 (catheters)	NR	NR	Long and short-acting opioid medications and muscle relaxer after discharge
Julien-Marsollier et al. 2020 [38]	Day 1 (physiotherapy)	Avoidance of continuous background morphine infusions with patient-controlled analgesia, opioid-sparing pharmacological and non-pharmacological techniques (cooling brace, 400 mg/day gabapentin for 5 days)	NR	Rapid feeding	Day 1 (drains), day 2 (catheters)	NR	IV fluid administration (balanced crystalloid solution at 2mlkg − 1 h − 1)	NR
Kalinin et al. 2021 [39]	First h after surgery (rehabilitation), day 1 (physiotherapy)	Opioid sparing multimodal analgesia, recovery from post-anesthetic depression	Within first 12 h after surgery	NR	No drain or removal at day 1, catheter removal in operating room	NR	Within first 12 h after surgery	NR
Kerolus et al. 2021 [40]	Day 0 and 1 (mobilization and aggressive physical therapy), cryotherapy (ice packs for 10-15 min 6 times per day, initiated in recovery room and for 72 h postoperatively)	Multimodal analgesia (acetaminophen 625 mg if pain score ≤ 3, hydrocodone-acetaminophen 5-325 mg if pain ≤4–6 every 4 h, hydrocodone-acetaminophen 10-325 mg if pain ≤7–10 every 4 h, tramadol 100 mg every 4 h, IV morphine 2-4 mg every 4-6 h, morphine patient-controlled analgesia, if necessary, pregabalin 75 mg)	Subcutaneous IV heparin at day 1	Early nutrition (1 L normal saline), bowel regimen (docusate-sodium, senna-docusate 1 tablet scheduled at night, polyethylene glycol 1 tablet scheduled daily)	Catheter removal if placed (> 400 cc, if occurs 3 times in a row)	NR	Fluid management	Social work and nurse specialist rounds
Kilic et al. 2019 [41]	Within 2 h	Opioid-sparing multimodal approach (acetaminophen used before an opioid analgesic, tramadol as rescue analgesia)	Low-molecular-weight heparin subcutaneously	Early oral intake	NR	NR	NR	NR
Kilic et al. 2020 [42]	Day 0 with movements 3 times daily	Opioid-sparing approach (acetaminophen and NSAIDs with pain > 4, tramadol with pain > 8)	Low-molecular-weight heparin	Early food and drink intake	NR	NR	NR	NR
Kim et al. 2021 [43]	Day 0 (physical therapy and rehabilitation)	Multimodal analgesia (ketorolac, acetaminophen, or IV hydromorphone patient-controlled analgesia for breakthrough pain at day 1), oral opioid, dexamethasone at day 2	Day 0 (pharmacologic prevention)	Early bowel function return	Day 1 (catheters), Day 3 (drains, output < 80 cc/8-h)	NR	Minimizing blood transfusions	NR
Lampilas et al. 2021 [44]	Day 0	Oral multimodal analgesia (NSAIDs, muscle relaxants)	NR	Oral nutrition	Day 0, at the end of intervention (catheter), day 1 (drain)	NR	NR	X-ray control, follow-up
Li et al. 2018 [45]	Day 1	NR	Deep vein ultrasound (for high-risk patients), pneumatic pump, stretch socks	Day 0 (early oral food intake, regular diet)	Day 1 (catheter), day 2 (drain)	NR	Less infusion volume (1000mlx2 days)	NR
Li et al. 2020 [46]	Within 4 h after surgery	Multimodal analgesia (no analgesia or oral minimal dose nonopioid with pain < 4, oral or IV nonopioid with pain 4–6, opioid with pain ≥7)	Limb movement, antithrombotic stockings	Early oral feeding	Catheter removal returning to the ward	NR	NR	NR
Li et al. 2021 [47]	Within 4 h after surgery	Multimodal analgesia (no analgesia or oral minimal dose nonopioid with pain < 4, oral or IV nonopioid with pain 4–6, opioid with pain ≥7)	Limb movement, antithrombotic stockings	Early oral feeding	Catheter removal returning to the ward	NR	NR	NR
Nazarenko et al. 2016 [48]	2 h after surgery (rehabilitation)	Short-acting anesthetics	NR	Enteral nutrition	NR	NR	NR	2–3 day after operation
Rao et al. 2021 [49]	Yes	Epidural infusion 0.1% ropivacaine 0.2 cc/kg/h (maximum rate 10 cc/h), hydromorphone patien-controlled analgesia (discontinued day 2), nonopioid and opioid analgesics (ketorolac, diazepam, gabapentinoids, acetaminophen, oral pregabalin, oral ossicodone, naloxone, oral ibuprofen)	NR	Early nutrition, clear liquid, bowel regimen, stool softeners, chewing gum	Day 2 (epidural catheter)	Antibiotics	NR	Follow-up (3–4 weeks), oral opioids, diazepam, valium, acetaminophen, ibuprofen, multivitamin
Shaw et al. 2021 [50]	NR	Patient-controlled analgesia morphine (0.02 mg/kg), oral hydrocodone and acetaminophen (5-10 mg/325 mg) every 4 h, ketorolac (0.5 mg/kg) every 6 h, 300 mg gabapentin (up to 3 times a day), diazepam (2 mg) every 4 h	NR	NR	NR	NR	NR	NR
Smith et al. 2019 [51]	Day 1 (early physical therapy and mobilization)	Non-opioid regimen for 7 days (celecoxib 200 mg every 12 h, gabapentin 300 mg every 8 h, acetaminophen 975 mg every 6 h)	NR	Diet, stool softeners, laxatives	Early catheter removal	NR	NR	Visit (2 weeks), follow-up
Soffin et al. 2019b [52]	Within 2 h	Opioid-sparing multimodal analgesia (acetaminophen, NSAIDs, gabapentin, tramadol)	NR	Early nutrition	NR	NR	IV fluid administration cessation	Short course of tramadol
Soffin et al. 2019a [53]	Within 2 h	Opioid-sparing multimodal analgesia (acetaminophen 1000 mg every 6 h, NSAIDs or nonpharmacological intervention with pain score < 5, meloxicam 7.5 mg every 12 h, oral tramadol 50 mg × 2 doses as needed with p’ain score ≥ 5, oxycodone 5 mg evary 3 h as needed with pain score ≥ 8)	NR	Early oral intake	NR	NR	NR	Follow-up plan
Soffin et al. 2019c [54]	Within 90 min	Non-opioid analgesics (acetaminophen, ketorolac, gabapentin, ice, position changes if pain ≤4, 2 50-mg doses tramadol if pain 5–7, 5-mg oxycodone if pain 8–10)	NR	Early feeding	NR	NR	NR	NR
Soffin et al. 2020 [55]	Within 2 h, physical therapy (twice daily)	Oral opioids (50-100 mg tramadol or 5-10 mg oxycodone), patient-controlled analgesia (0.2 mg·ml-1 hydromorphone IV every 10 min, 1000 mg IV and oral acetaminophen every 6 h), opioid-sparing multimodal analgesia (15-30 mg ketorolac every 8 h, 300 mg gabapentin every 8 h, 45 mg dextromethorphan every 8 h)	Pneumatic compression devices	Early oral intake (fluid and solid), bowel regimen (constipation and ileus prevention)	NR	NR	NR	NR
Staartjes et al. 2019 [56]	2 h after surgery, physical therapy	Opioid-sparing analgesia (NSAIDs, paracetamol, patient-controlled analgesia w/short-acting opioids)	Low-molecular-weight heparin	Day 0 (early solids and fluids intake)	Early drains and catheters removal	NR	NR	Telephone call (2–14 days after surgery), clinical and radiological follow-up (6 weeks)
Venkata et al. 2018 [57]	Day 1	Multimodal analgesia	NR	NR	NR	NR	NR	NR
Wang et al. 2017 [58]	Day 0 or 1	Analgesia (gabapentin, tramadol, acetaminophen)	Stockings and intermittent pneumatic compression	Oral nutrition	NR	NR	NR	Day 1 (audit, radiographs)
Wang et al. 2020 [59]	Yes	Multimodal analgesia	Yes (stockings)	Early oral feeding, gastrointestinal management	Early catheter removal	NR	NR	NR
Yang et al. 2020a [60]	Day 1	Patient-controlled analgesia (discontinued at day 1), oral pain medication (oxycodone, valium, neurontin for 5 days, Tylenol, toradol)	NR	High fiber diet (day 1)	NR	NR	NR	Day 2–5, visit (2 weeks after)
Yang et al. 2020b [61]	Rehabilitation	NSAID intramuscularly, IV or orally, tramadol if necessary	Intermittent pneumatic compression within several h following the end of operation, compression stocking for 1 week, muscle contraction exercise in the bed	Diet recovery (liquid diet < 200 mL 2 h after surgery, eating and drinking day 1)	Day 1 (drains and catheters)	Infection prevention (second-generation cephalosporins prophylactic use restricted within 24 h after the end of surgery, and advanced broad-spectrum antibiotics if necessary)	NR	NR
Yang et al. 2021 [62]	Day 1–2	Multimodal analgesia (local subcutaneous anesthetics before closure with 0.75% ropivacaine 10 mL + 0.9% saline 10 mL, patient-controlled analgesia with sufentanil 100 mg + butorphanol 8 mg + 0.9% saline, IV parecoxib 40 mg day 1, oral analgesics day 2, celecoxib capsule 200 mg or etoricoxib tablets 120 mg, once daily, with eperisone hydrochloride tablets 50 mg, 3 times daily)	NR	Early oral intake (clear liquid after 2 h, soft diet 4-6 h, normal diet day 2)	Within 24 h (subcutaneous drainage, < 50 mL daily)	NR	NR	NR
Young et al. 2021 [63]	Within 12 h	Multimodal analgesia (acetaminophen and gabapentin, celecoxib in nonfusion cases), opioids	Subcutaneous heparin injections (anticoagulation)	Bowel regimen, regular diet	Day 1 (catheter)	Yes	NR	NR
Band et al. 2022 [64]	Yes	Baclofen 10 mg orally 3 times daily as needed, gabapentin 300 mg every night at bedtime, and acetaminophen 650 mg every 4-6 h as needed	NR	NR	Day 1 (drain), day 0 (catheter)	NR	NR	Gabapentin, baclofen, acetaminophen, and opioids only if necessary
Chen et al. 2022 [65]	Yes	Multimode analgesia (IV ketoprofen acid, oral celecoxib, and im dezocine 5 mg if necessary)	Low-molecular-weight heparin calcium, intermittent pneumatic compression	Oral feeding	Day 1 (drain), day 1–2 (catheter)	NR	NR	Satisfaction survey, multiple daily visits
Leng et al. 2022 [66]	Day 1	Opioid sparing, IV parecoxib 40 mg, celecoxib 200 mg and pregabalin 75 mg every 12 h, tramadol 100 mg if necessary	NR	Oral diet, clear liquids (day 0)	Day 1 (catheter), day 2 (drain)	NR	NR	Mobile app, NRS, NDI and JOA scores
Porche et al. b2022 [67]	Day 0–1	Multimodal analgesia (IV acetaminophen, duloxetine or gabapentin, methadone), narcotics, no valium and NSAIDs, baclofen or cyclobenzaprine for spasms	Subcutaneous heparin (5000 − 7500 U/kg, day 0), lower extremity pneumatic pumps, on-bed movement, and early off-bed mobilization	Diet ordered and protein supplements (day 0), scheduled polyethylene glycol, docusate, and senna	Day 0 (catheter), day 1 (drain)	NR	IV fluids discontinuation (day 1)	NR
Porche et al. a2022 [68]	Day 0–1	Multimodal analgesia (IV acetaminophen, duloxetine or gabapentin, methadone), narcotics, no valium and NSAIDs, baclofen or cyclobenzaprine for spasms	Subcutaneous heparin (5000 − 7500 U/kg, day 0), lower extremity pneumatic pumps, on-bed movement, and early off-bed mobilization	Diet ordered and protein supplements (day 0), scheduled polyethylene glycol, docusate, and senna	Day 0 (catheter), day 1 (drain)	NR	IV fluids discontinuation (day 1)	NR
Sun et al. 2022 [69]	Yes (functional exercise)	NR	NR	Diet	NR	NR	NR	Follow-up phone call (VAS, BI and ODI scores)
Wang et al. 2022 [70]	Physical therapy within 2 h, ambulating within 48 h	Multimodal analgesia	NR	Early drinking water, early feeding 6 h after	Catheter (after 24 h)	NR	NR	NR
Zhang et al. 2022 [71]	Yes (4, 8 and 24 h after)	Analgesia (parecoxib sodium and morphine, oral etoricoxib)	Physical cold and adjustable negative pressure suction	Oral diet (day 2)	NR	NR	NR	NR

*Abbreviations*: *Ref* references, *DVT* deep venous thrombosis, *yr* years, *NR* not reported, *TXA* tranexamic acid, *PRBC* packed red blood cells, *Hb* hemoglobin, *min* minutes, *IV* intravenous, *h* hours, *PRN* Pro re nata, *CT* computed tomography, *MRI* Magnetic resonance imaging, *GDHM* Goal directed hemodynamic management, *PPV* pulse pressure variability, *SVV* stroke volume variability, *CO* cardiac output, *NSAIDs* nonsteroidal anti-inflammatory drugs, *BMI* Body Mass Index, *LIA* local infiltration analgesia, *MED* morphine equivalent dose, *TIVA* Total intravenous anesthesia, *EBL* estimated blood loss, *ICU* intensive care unit, *MME* milligram morphine equivalents, *PONV* postoperative nausea and vomiting, *MRSA* methicillin resistant *Staphylococcus aureus*, *ABG* arterial blood gas, *TEG* thromboelastography

### Assessment of methodological quality

The methodological quality of selected studies was independently assessed by two reviewers (DC and FS), using the Quality Assessment Tools of the National Heart, Lung, and Blood Institute (NHLBI) [72]. The tool included 14 items, which assessed the possible sources of bias. For each item, we categorized “Yes” if the criterion was explicitly met, “No” if the assessed criterion was not met. In case of disagreement, the reviewers attempted to reach consensus by discussion; if this failed, a third reviewer (MF) was consulted making the final decision.

## Results

### Study selection and characteristics

**Fig. 1:**
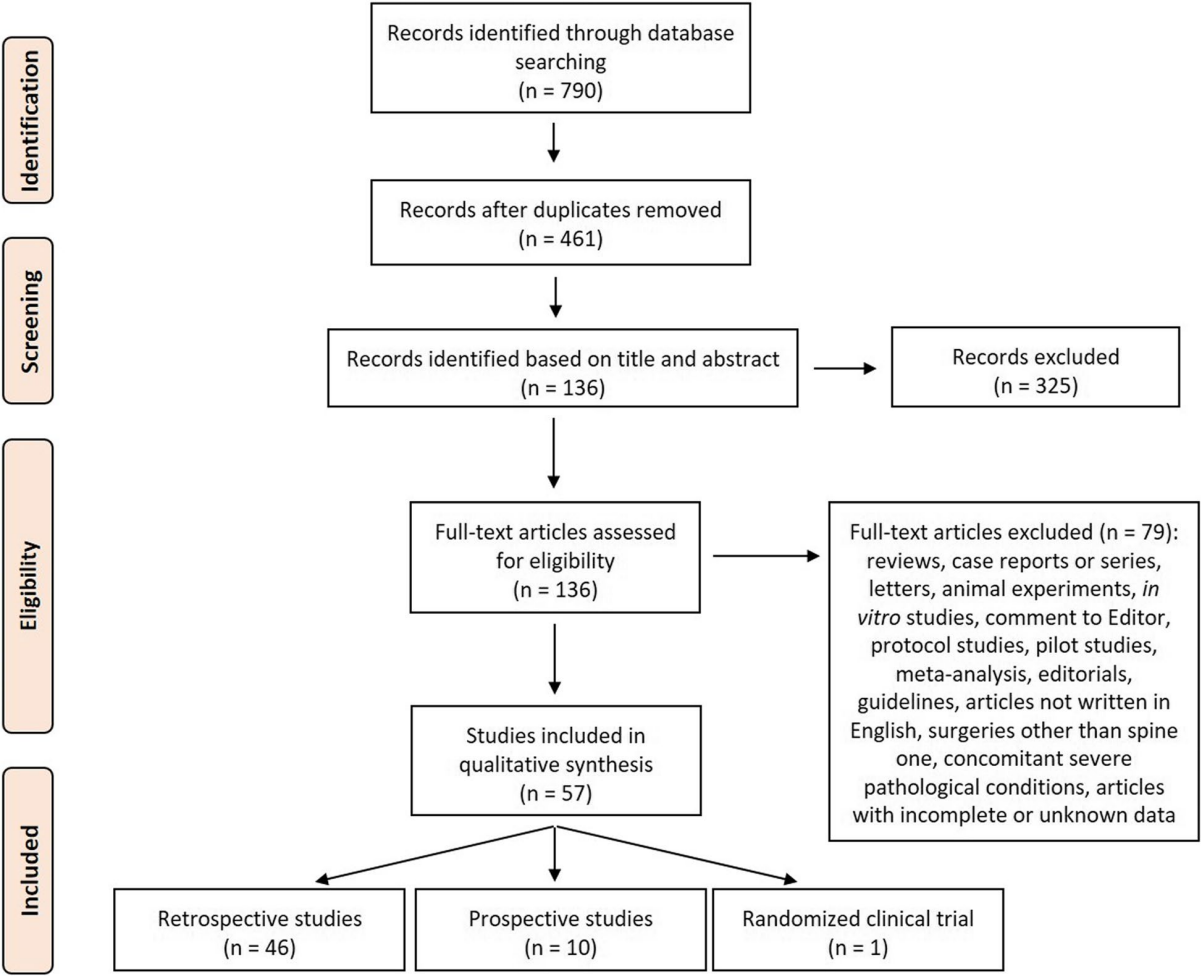
PRISMA flow diagram for the selection of studies

The initial literature search retrieved 790 studies. Of those, 328 studies were identified using PubMed, 266 using Scopus, 196 were found in Web of Science Core Collection. Subsequently, articles were submitted to a public reference manager to eliminate duplicate. The resulting 461 articles were screened for title and abstract and 136 articles were reviewed to establish whether the publication met the inclusion criteria. Finally, 57 articles were considered eligible for this review. Search strategy and study inclusion and exclusion criteria are detailed in Fig. 1. Of these articles, 46 were retrospective cohort studies, 10 were prospective cohort studies and 1 was randomized clinical trial (RCT).

### Assessment of methodological quality

In our quality assessment for spine surgery the 44% [15, 23, 27, 29, 32–38, 40–42, 46–49, 51, 55, 56, 61, 67, 68, 70] of the studies were rated strong, 25% [17, 18, 21, 22, 25, 28, 31, 39, 43, 44, 60, 62, 63, 65] were rated moderate, and 32% [16, 19, 20, 24, 26, 30, 45, 50, 52–54, 57–59, 64, 66, 69, 71] were rated weak. Methodological weaknesses that led to moderate or weak quality scores often included the lack of a sample size justification, power description, or variance and effect estimates, the lack of subjects selected or recruited from the same population, the lack of results evaluation more than once over in time, the lack of blinded assessor and the lack of measurement of potential confounding variables. Risks of bias assessments for each study were summarized in Table 3.

**Table 3:** National Heart, Lung, and Blood Institute (NHLBI) quality assessment tool

Reference	Criteria													
	1	2	3	4	5	6	7	8	9	10	11	12	13	14
Adeyemo et al. 2021a [15]	Y	Y	Y	N	N	Y	Y	Y	Y	Y	Y	N	Y	N
Adeyemo et al. 2021b [16]	Y	Y	Y	N	N	N	Y	Y	N	N	Y	N	Y	N
Angus et al. 2019 [17]	Y	Y	Y	N	N	N	Y	Y	Y	N	Y	N	Y	Y
Brusko et al. 2019 [18]	Y	Y	Y	Y	N	N	Y	Y	N	Y	Y	N	Y	N
Carr et al. 2019 [19]	Y	Y	Y	N	N	N	Y	Y	Y	N	Y	N	Y	N
Chang et al. 2020 [20]	Y	Y	Y	N	N	N	Y	Y	N	Y	Y	N	Y	N
Chen et al. 2021 [21]	Y	Y	Y	Y	N	Y	Y	N	Y	N	Y	N	Y	N
Dagal et al. 2019 [22]	Y	Y	Y	Y	N	N	Y	Y	N	N	Y	N	Y	Y
d’Astorg et al. 2020 [23]	Y	Y	Y	Y	N	Y	Y	Y	Y	N	Y	N	Y	Y
Debono et al. 2019 [24]	Y	Y	Y	N	N	N	Y	Y	Y	N	Y	N	Y	N
Debono et al. 2021 [25]	Y	Y	Y	N	Y	N	Y	Y	Y	N	Y	N	Y	N
DeVries et al. 2020 [26]	Y	Y	Y	N	N	N	Y	N	N	N	Y	N	Y	N
Duojun et al. 2021 [27]	Y	Y	Y	Y	N	N	Y	Y	Y	Y	Y	N	Y	N
Feng et al. 2019 [28]	Y	Y	Y	Y	N	N	Y	Y	Y	N	Y	N	Y	N
Flanders et al. 2020 [29]	Y	Y	Y	N	Y	N	Y	Y	Y	Y	Y	N	Y	N
Fletcher et al. 2020 [30]	Y	Y	Y	Y	N	Y	Y	N	N	N	Y	N	Y	N
Fletcher et al. 2021 [31]	Y	Y	Y	Y	N	N	Y	Y	N	N	Y	N	Y	Y
Garg et al. 2021 [32]	Y	Y	Y	N	Y	N	Y	Y	Y	Y	Y	N	Y	N
Gong et al. 2021 [33]	Y	Y	Y	Y	N	Y	Y	Y	Y	Y	Y	Y	Y	N
He et al. 2020 [34]	Y	Y	Y	Y	Y	Y	Y	Y	N	N	Y	Y	Y	N
Heo et al. 2019 [35]	Y	Y	Y	Y	N	Y	Y	Y	Y	Y	Y	N	N	N
Ifrach et al. 2020 [36]	Y	Y	Y	N	Y	N	Y	Y	Y	Y	Y	N	Y	N
Jazini et al. 2021 [37]	Y	Y	Y	Y	N	Y	Y	Y	Y	N	Y	N	Y	Y
Julien-Marsollier et al. 2020 [38]	Y	Y	Y	N	Y	N	Y	Y	Y	Y	Y	N	Y	Y
Kalinin et al. 2021 [39]	Y	Y	Y	Y	N	N	Y	Y	Y	N	Y	N	Y	N
Kerolus et al. 2021 [40]	Y	Y	Y	Y	Y	N	Y	Y	Y	Y	Y	N	Y	N
Kilic et al. 2019 [41]	Y	Y	Y	Y	N	Y	Y	Y	Y	Y	Y	N	Y	N
Kilic et al. 2020 [42]	Y	Y	Y	Y	N	Y	Y	Y	Y	Y	Y	N	Y	N
Kim et al. 2021 [43]	Y	Y	Y	N	Y	N	Y	Y	Y	N	Y	N	Y	N
Lampilas et al. 2021 [44]	Y	Y	Y	Y	N	N	Y	Y	Y	N	Y	N	Y	N
Li et al. 2018 [45]	Y	Y	Y	N	N	N	Y	Y	Y	N	Y	N	Y	N
Li et al. 2020 [46]	Y	Y	Y	Y	Y	Y	Y	N	Y	N	Y	N	Y	N
Li et al. 2021 [47]	Y	Y	Y	Y	N	Y	Y	Y	Y	Y	Y	N	Y	N
Nazarenko et al. 2016 [48]	Y	Y	Y	Y	N	Y	Y	Y	Y	Y	Y	N	Y	N
Rao et al. 2021 [49]	Y	Y	Y	Y	Y	N	Y	Y	Y	N	Y	N	Y	N
Shaw et al. 2021 [50]	Y	Y	N	Y	Y	N	Y	Y	N	N	Y	N	Y	N
Smith et al. 2019 [51]	Y	Y	Y	Y	Y	N	Y	Y	Y	N	Y	N	Y	N
Soffin et al. 2019a [53]	Y	Y	Y	Y	N	N	Y	N	Y	N	Y	N	Y	N
Soffin et al. 2019b [52]	Y	Y	Y	Y	N	N	Y	N	Y	N	Y	N	Y	N
Soffin et al. 2019c [54]	Y	Y	Y	Y	N	N	Y	N	Y	N	Y	N	Y	N
Soffin et al. 2020 [55]	Y	Y	Y	Y	Y	N	Y	Y	Y	Y	Y	Y	Y	N
Staartjes et al. 2019 [56]	Y	Y	Y	Y	Y	N	Y	N	Y	Y	Y	N	Y	N
Venkata et al. 2018 [57]	Y	Y	Y	Y	N	N	Y	N	N	N	Y	N	Y	N
Wang et al. 2017 [58]	Y	Y	Y	Y	N	N	Y	N	Y	N	Y	N	Y	N
Wang et al. 2020 [59]	Y	Y	Y	Y	N	N	Y	N	Y	N	Y	N	Y	N
Yang et al. 2020a [60]	Y	Y	Y	Y	Y	N	Y	N	Y	N	Y	N	Y	N
Yang et al. 2020b [61]	Y	Y	N	Y	N	Y	Y	Y	Y	Y	Y	Y	Y	N
Yang et al. 2021 [62]	Y	Y	Y	N	N	Y	Y	Y	Y	N	Y	N	Y	N
Young et al. 2021 [63]	Y	Y	Y	N	Y	Y	Y	N	Y	N	Y	N	Y	N
Band et al. 2022 [64]	Y	N	Y	N	N	Y	N	Y	Y	N	Y	N	Y	N
Chen et al. 2022 [65]	Y	Y	Y	N	N	Y	Y	Y	Y	N	Y	N	Y	N
Leng et al. 2022 [66]	Y	Y	Y	N	N	N	Y	Y	Y	N	Y	N	Y	N
Porche et al. b2022 [67]	Y	Y	Y	Y	Y	Y	Y	Y	Y	N	Y	N	Y	Y
Porche et al. a2022 [68]	Y	Y	Y	N	Y	Y	Y	Y	Y	N	Y	N	Y	Y
Sun et al. 2022 [69]	Y	Y	Y	Y	N	Y	N	N	Y	N	Y	N	Y	N
Wang et al. 2022 [70]	Y	Y	Y	Y	N	Y	Y	Y	Y	N	Y	N	Y	N
Zhang et al. 2022 [71]	Y	Y	Y	N	N	Y	N	Y	Y	N	Y	N	Y	N

1. Was the research question or objective in this paper clearly stated? 2. Was the study population clearly specified and defined? 3. Was the participation rate of eligible persons at least 50%? 4. Were all the subjects selected or recruited from the same or similar populations (including the same time period)? Were inclusion and exclusion criteria for being in the study prespecified and applied uniformly to all participants? 5. Was a sample size justification, power description, or variance and effect estimates provided? 6. For the analyses in this paper, were the exposure(s) of interest measured prior to the outcome(s) being measured? 7. Was the timeframe sufficient so that one could reasonably expect to see an association between exposure and outcome if it existed? 8. For exposures that can vary in amount or level, did the study examine different levels of the exposure as related to the outcome (e.g., categories of exposure, or exposure measured as continuous variable)? 9. Were the exposure measures (independent variables) clearly defined, valid, reliable, and implemented consistently across all study participants? 10. Was the exposure(s) assessed more than once over time? 11. Were the outcome measures (dependent variables) clearly defined, valid, reliable, and implemented consistently across all study participants? 12. Were the outcome assessors blinded to the exposure status of participants? 13. Was loss to follow-up after baseline 20% or less? 14. Were key potential confounding variables measured and adjusted statistically for their impact on the relationship between exposure(s) and outcome(s)?

Y Yes, N No

### Studies results

#### General information’s

A total of 11,385 (with a range from 17 to 2579) and 6040 (with a range from 15 to 1563) patients were analyzed for the fast-track and non-fast-track groups (traditional protocol) respectively. Mean age of the patients was 52 years (with range from 13.2 ± 3.2 to 76.68 ± 4.83) for fast-track group, and 54 (with range from 14.3 ± 1.9 to 7 6.38 ± 4.48) for non-fast-track group. Most of the patients were women (8515) compared to men (8171). In addition, 8 studies analyzed adolescent patients under the age of 18.

#### Types of spine surgery and pathological conditions

Procedures associated with the spine included minor, major, and complex surgeries, such as arthrodesis, corpectomy, microdiscectomy, decompression, laminectomy, laminoplasty, open and minimally invasive posterior lumbar interbody fusion (PLIF), transforaminal lumbar interbody fusion (TLIF), oblique lumbar interbody fusion (OLIF), anterior lumbar interbody fusion (ALIF), anterior cervical discectomy and fusion (ACDF), percutaneous endoscopic transforaminal discectomy (PETD), percutaneous endoscopic lumbar interbody fusion (PELIF), and cervical disc arthroplasty (CDA). Because the types of spine surgery were not standardized across the 57 studies, it was difficult to quantify the prevalence of any single type of spine procedure among the fast-track protocols. However, most fast-track protocols were implemented for lumbar spine procedure, mainly through techniques such as PLIF and TLIF and at the spinal levels L1-L5. In addition, of the 57 articles included in this review, 81% presented a comparison with a standard/traditional protocol (non-fast-track) while the others (19%) evaluated different fast-track protocols in patients undergoing spine surgery. It was shown that fast-track programs were applied to different spine diseases, mainly for degenerative pathological conditions as disc herniation, stenosis, spondylolysis, radiculopathy, spondylolisthesis (78%), for adult spinal deformity (5%) or both (3%). In addition,, a total of 1200 patients (8 studies, 14%) were treated for adolescent idiopathic scoliosis using a posterior approach. Of these 8 studies, 6 were retrospective, while the other 2 were prospective. Concerning adult deformities, they were evaluated in 3 retrospective studies, using an anterior or posterior approach. Finally, 45 studies evaluated patients treated for degenerative diseases, using an anterior or posterior approach. Of these, 36 studies were retrospective, 8 studies were prospective, and only one study was a RCTs.

#### Comorbidity

Several comorbidities such as osteoporosis, frailty, obesity, sarcopenia, hypertension, diabetes, chronic cardiovascular disease, chronic obstructive pulmonary disease, obstructive sleep apnea, chronic kidney and liver diseases, depression and dementia were reported in 30 studies.

#### Components of fast-track procedures in spine surgery

##### Preoperative

In this review, one of the principal preoperative interventions was patient education and information (detailed information provided to the participants and their family members regarding the surgical procedure, potential complications, rehabilitation, and hospital discharge) (86%), followed by multidisciplinary consultations (geriatric, psychological, nutritional, behavioral health) to guide patients’ expectations as well as to inform them on the risks about intra- and postoperative pathway (61%), nutrition (minimized fasting) (61%), pain management (analgesic drugs use) (33%) and physical therapy (21%).

##### Intraoperative

In the studies examined, principal intraoperative components were multimodal analgesia and pain management (82%), antimicrobial/antibiotic prophylaxis (44%), normothermia/normovolemia maintenance (53%), and general anesthesia (42%). Additionally, nausea and vomiting prevention (with antiemetics or compression hosiery) (37%), carbohydrate loading 4 hours before surgery and clear fluid and solid fluid fasting for 2–6 hours before surgery (42%), transfusion control (28%), tranexamic acid (TXA) use (including oral and parenteral formulations) as strategy to minimize bleeding (33%) and the avoidance of catheter/drain (22%) represented the key fast-track interventions in spine surgery.

##### Postoperative

The principal postoperative elements were pain management with multimodal analgesia use (89%), and early mobilization within 24 hours with rehabilitation/physiotherapy (72%). Other common elements were early nutrition and bowel regime maintenance (74%), catheter/drain removal within 24 hours after surgery (54%) and thromboprophylaxis (37%). Finally, normovolemia maintenance (16%) and antimicrobial/antibiotic prophylaxis (21%) were others postoperative key element.

#### Length of hospital stay, complication and readmission rates

The primary outcome in fast-track studies on spine surgery was LOS. A LOS of 2–5 days for the spinal deformities, such as adolescent idiopathic scoliosis was observed, while a LOS of 2–12 days were detected for the degenerative diseases as disc herniation, stenosis, spondylolysis, radiculopathy and spondylolisthesis. Most studies evaluated a fast-track protocol (81%) versus a conventional (non-fast track) protocol (19%), reporting a significantly reduced LOS (in 81% of studies), without increasing complication or readmissions rate in patients treated with fast-track, regardless of follow-up (from 1 month to 2 years), pathology and surgical approach used. Four studies instead reported no significant differences in LOS between the fast-track and non-fast-track groups [16, 33, 51, 53]. Complication rates with fast-track protocols ranged from 1.5 to 26%. The most reported complications were urinary retention, constipation, motor block, arrhythmia, pneumonia, wound infection/dehiscence, neurological deficit, pain, nausea and vomiting; however, no studies reported an increase in complications associated with fast-track protocols. Conversely, a decrease in adverse events and readmissions associated with the fast-track protocol has been reported in 25% of studies. Only in a few cases was performed a revision surgery mostly for traumatic fracture, screw misplacement and/or removal, graft migration, cerebrospinal fluid leakage, unmanageable pain, and wound infection.

### Outcomes and clinical evidence of fast-track protocols

In 46% of studies, general anesthesia by an endotracheal intubation with total intravenous anesthesia (TIVA), using mostly propofol and ketamine, was the usual procedure adopted for spine surgery [15, 19, 20, 32, 34, 35, 37–42, 46, 47, 52–58, 61, 62, 64, 67, 68]. Subsequently, for maintenance of anesthesia, inhalational agents such as sevoflurane, isoflurane and desflurane, or intravenous opioid agents such as sufentanil or remifentanil infusions were used. In 21% of studies, a local anesthesia with anesthetic agents as bupivacaine, lidocaine or ropivacaine was employed [18, 27, 28, 33, 35, 45, 48, 63, 65, 66, 70, 71]. Regarding analgesia management, pain scores were tracked in 30 studies. A significant reduction in pain through visual analog scale (VAS) score, was observed with the fast-track protocols in 40% of studies [18, 21, 23, 27, 31–33, 35–39, 41, 42, 45, 47, 48, 55, 60–62, 65, 71]. The pain reduction during fast-track pathways were associated to pre-emptive and postoperative analgesia use and to intraoperative local analgesics infiltration (LIA). Several opioid-sparing agents at different concentrations were used for pain relief, specifically, the most used are acetaminophen (1000 mg), gabapentin (300 or 600 mg), pregabalin (75 mg), celecoxib (200 mg) and non-steroidal anti-inflammatory drugs (NSAIDs). Studies demonstrated that this analgesic protocol not only reduced the opioid requirements but also helped to reduce post-operative nausea-vomiting and the risk of complications. Five studies reported little or no difference in pain scores between fast-track and non-fast-track groups, despite a decrease in opioid use after surgery (28%) was detected. A reduction in intraoperative blood loss (25%) and in transfusion rates (5%) with fast-track protocols vs. non-fast-track protocols were also seen in several studies; this aspect is due to the prevention of blood loss and transfusion protocols control as well as thromboprophylaxis (compression stockings or low molecular weight heparin use), maintaining of the body temperature (at 36°-37°, with hot air blanket, fluid warmers or convective warming device) and of fluids and blood pressure. The main blood-saving strategy applied in this review is the TXA use in intraoperative phase, mostly at 10 mg/kg concentration and administered intravenously, orally or topically (1 g). The TXA is an antifibrinolytic medication that stops the breakdown of fibrin clot by inhibiting activation of plasminogen, plasmin, and tissue plasminogen activator. On the other hand, transfusion protocols included control of hemoglobin, platelet and fibrinogen parameters. In addition, a reduction in intraoperative time (19%), catheters and drains removal time (19%), and total health costs (10%) were also detected in these studies. The fast-track elements not only improved the treatment management of the patients, increasing their satisfaction, but also helped the range of motion and return of normal function in all the examined studies that evaluated these parameters (9%). Postoperatively, physiotherapy was applied to increase the range of motion and enhance gait. The improvement in motion and return of function was undoubtedly helped by the early mobilization, by the early nutrition but also by pain management as well as by the information and support given to the patients by the interdisciplinary team, as it has increased their sense of security and satisfaction.

## Conclusion

Despite the increasing rates of spine procedures, standardized criteria for pre-, intra- and postoperative management for specific surgeries are lacking. Given the apparent benefits of fast-track programs in other surgical disciplines, implementation of these protocols in spine surgery could be of key importance to reduce LOS, accelerate return of function, minimize postoperative pain, and save costs. Notwithstanding the presence of several preliminary cohort studies that lack of control groups and showed a variability in operations, surgical indications and pathological spine diseases, most reviewed studies demonstrated that fast-track pathways in spine surgery are associated with a shorter LOS and accelerated return to function without increasing rates of complications or readmissions. Furthermore, it was shown that although several of the analyzed fast-track protocols differed in the exact analgesic regimen, the multimodal pain control was a common feature. Given the broad side effect profile of opioid drugs, the use of additional analgesics where possible is encouraged. Another key point of fast-track protocols were the use of TXA, administered either intravenously or orally, that almost eliminated the need for other blood conservation strategies. The reviewed studies also evidenced that early oral intake after surgery is safe and can accelerate the restoration of bowel function and shorten the LOS. Another benefit is that of early mobilization after spinal surgery that led to a reduced rates of infections and medical complications along with a further decrease in mean LOS. In addition to accelerating the return to basic functional level, accelerated walking and rehabilitation also serve to emphasize the patient’s role in recovery.

Despite these promising results, currently, it is difficult to isolate the effect of fast-track elements on patient outcome. It is also difficult to determine whether fast-track would be more successful for specific spine surgeries or pathologies. In fact, current literature for fast-track spinal deformities and AIS is restricted to few clinical studies that are manly retrospective studies with non-randomized data, and initial cohort studies. Furthermore, the different spine procedures vary in expected surgical stress levels and recovery rates, as do the age and patients comorbidity and, to date, these distinctions have not yet been made. Based on these limitations, larger RCTs are mandatory, especially for patients with spinal deformities, to provide robust evidence and establish the efficacy of enhanced-recovery programs for patient populations and procedures within orthopedic spine surgery. Finally, the need for a specific and uniform evidence-based protocol is important to enhance both patient and process outcomes.

## Supplementary Information

**Additional file 1:** **Table 1.** Search terms used in the PubMed, Scopus, and Web of Science Core Collection.

## Data Availability

The datasets used and/or analysed during the current study are available from the corresponding author on reasonable request.

## Abbreviations

LOS: Length of stay
ERAS: Enhanced Recovery After Surgery
PICOS: Population, intervention, comparison, outcomes, study design
PRISMA: Preferred Reporting Items for Systematic Reviews and Meta-Analyses
ICU LOS: Intensive care unit length of stay
DVT: Deep venous thrombosis
NHLBI: National Heart, Lung, and Blood Institute
RCT: Randomized clinical trial
PLIF: Posterior lumbar interbody fusion
TLIF: Transforaminal lumbar interbody fusion
OLIF: Oblique lumbar interbody fusion
ALIF: Anterior lumbar interbody fusion
ACDF: Anterior cervical discectomy and fusion
PETD: Percutaneous endoscopic transforaminal discectomy
PELIF: Percutaneous endoscopic lumbar interbody fusion
CDA: Cervical disc arthroplasty
TXA: Tranexamic acid
TIVA: Total intravenous anesthesia
VAS: Visual analog scale
LIA: Local analgesics infiltration
NSAIDs: Non-steroidal anti-inflammatory drugs.
