# Supplementary material for: Fast-track protocols for patients undergoing spine surgery: a systematic review
Source: BMC Musculoskelet Disord. 2023 Jan 23;24:57. doi: 10.1186/s12891-022-06123-w (PMC9869597; doi:10.1186/s12891-022-06123-w)
Supplement: Supplementary file 1 — Additional file 1: Table 1. Search terms used in the PubMed, Scopus, and Web of Science Core Collection. [file 12891_2022_6123_MOESM1_ESM.docx]

**Table 1:** Search terms used in the PubMed, Scopus, and Web of Science Core Collection.

| **Database** | **Free-vocabulary and/or Medical Subject Headings (MeSH) terms** |
| --- | --- |
| PubMed | (("spinal diseases"[MeSH Terms] OR ("spinal"[All Fields] AND "diseases"[All Fields]) OR "spinal diseases"[All Fields] OR ("spine"[All Fields] AND "disease"[All Fields]) OR "spine disease"[All Fields] OR (("spine"[MeSH Terms] OR "spine"[All Fields] OR "spines"[All Fields] OR "spine s"[All Fields]) AND ("surgery"[MeSH Subheading] OR "surgery"[All Fields] OR "surgical procedures, operative"[MeSH Terms] OR ("surgical"[All Fields] AND "procedures"[All Fields] AND "operative"[All Fields]) OR "operative surgical procedures"[All Fields] OR "general surgery"[MeSH Terms] OR ("general"[All Fields] AND "surgery"[All Fields]) OR "general surgery"[All Fields] OR "surgery s"[All Fields] OR "surgerys"[All Fields] OR "surgeries"[All Fields]))) AND ("fast-track"[All Fields] OR ("enhanced recovery after surgery"[MeSH Terms] OR ("enhanced"[All Fields] AND "recovery"[All Fields] AND "after"[All Fields] AND "surgery"[All Fields]) OR "enhanced recovery after surgery"[All Fields]) OR (("enhance"[All Fields] OR "enhanced"[All Fields] OR "enhancement"[All Fields] OR "enhancements"[All Fields] OR "enhancer"[All Fields] OR "enhancer s"[All Fields] OR "enhancers"[All Fields] OR "enhances"[All Fields] OR "enhancing"[All Fields]) AND ("recoveries"[All Fields] OR "recovery"[All Fields]) AND ("program"[All Fields] OR "program s"[All Fields] OR "programe"[All Fields] OR "programed"[All Fields] OR "programes"[All Fields] OR "programing"[All Fields] OR "programmability"[All Fields] OR "programmable"[All Fields] OR "programmably"[All Fields] OR "programme"[All Fields] OR "programme s"[All Fields] OR "programmed"[All Fields] OR "programmer"[All Fields] OR "programmer s"[All Fields] OR "programmers"[All Fields] OR "programmes"[All Fields] OR "programming"[All Fields] OR "programmings"[All Fields] OR "programs"[All Fields])))) AND ((y_10[Filter]) AND (english[Filter])) |
| Web of Science Core Collection | (TS = spine disease OR TS = spine surgery) AND (TS = fast-track OR TS = enhanced recovery after surgery OR TS = enhanced recovery programs) - with Publication Year from 2012 to 2022 |
| Scopus | (TITLE-ABS-KEY (spine AND disease) OR TITLE-ABS-KEY (spine AND surgery) AND TITLE-ABS-KEY (fast-track) OR TITLE-ABS-KEY (enhanced AND recovery AND after AND surgery) OR TITLE-ABS-KEY (enhanced AND recovery AND programs)) AND PUBYEAR > 2010 |
